# Large contributions of petrogenic and aged soil-derived organic carbon to Arctic fjord sediments in Svalbard

**DOI:** 10.1038/s41598-023-45141-z

**Published:** 2023-10-20

**Authors:** Dahae Kim, Jung-Hyun Kim, Youngkyu Ahn, Kwangchul Jang, Ji Young Jung, Minji Bae, Seung-Il Nam

**Affiliations:** 1 Korea Polar Research Institute, 26 Songdomirae-ro, Yeonsu-gu, Incheon, 21990 South Korea; 2 Department of Marine Science and Convergence Technology, Hanyang University ERICA Campus, 55 Hanyangdaehak-ro, Sangnok-gu, Ansan-si, Gyeonggi-do 15588 South Korea; 3 Department of Marine Science, Inha University, 100 Inha-ro, Michuhol-gu, Incheon, 22212 South Korea

**Keywords:** Biogeochemistry, Climate sciences, Environmental sciences, Ocean sciences

## Abstract

Svalbard fjords are recognized as hotspots for organic carbon (OC) burial and storage due to their high sedimentation rates, which effectively trap terrestrial sediments and inhibit extensive OC remineralization. In this study, we investigated surface sediments (n = 48) from eight Svalbard fjords, along with bedrock (n = 17), soil (n = 28), and plant (n = 12) samples, to identify the sources of sedimentary OC in these fjords using geochemical parameters. All examined surface sediments from the fjords showed a depletion in ^14^C_org_ (− 666.9 ± 240.3‰), indicating that recently fixed terrestrial and marine biomass alone cannot account for the entire sedimentary OC pool. Conventional bulk indicators such as N_org_/TOC ratio and δ^13^C_org_ were insufficient for fully determining the sources of sedimentary OC. Therefore, we employed a four-end-member approach, using Δ^14^C_org_, δ^13^C_org_, and lignin phenols to assess the relative contributions of petrogenic, soil-derived, plant-derived, and marine OC to the sedimentary OC pool. The analyzed fjord sediments consisted, on average, of 59.0 ± 28.1% petrogenic OC, 16.8 ± 12.1% soil-derived OC, 2.5 ± 2.2% plant-derived OC, and 21.8 ± 18.5% marine OC. This approach highlights the substantial contributions of petrogenic and aged soil-derived OC to present-day sedimentary OC in Svalbard fjords. Considering predicted global warming, accelerated inputs of petrogenic and soil-derived OC into fjords due to rapid glacier retreat may significantly impact the active carbon cycle and potentially contribute to CO_2_ emissions to the atmosphere, depending on burial efficiency.

## Introduction

In recent decades, the ‘land-to-ocean aquatic continuum’ between ‘terra firme’ terrestrial ecosystems and the open ocean has received considerable attention as aquatic critical zones in regulating the global carbon cycle on annual to centennial timescales^1,2^. Among these aquatic critical zones, fjords, mainly located in the mid-high latitudes, represent a relatively small fraction of the global continental margin (< 0.1%)^3^. Nevertheless, they serve as biogeochemical hotspots that effectively trap and store substantial amounts of organic carbon (OC)^1,4,5^. Annually, approximately 18 million tons of marine and terrigenous OC are buried in glacially depressed basins within fjords, accounting for approximately 11% of the global marine OC burial^6^ and 17 ± 12% of the global terrestrial OC burial^7^. As a result, the OC burial per unit area in fjords largely exceeds the global ocean average, making them a sensitive system for sequestering CO_2_ and regulating the global carbon cycle over geological time^1,6^.

**Figure 1:**
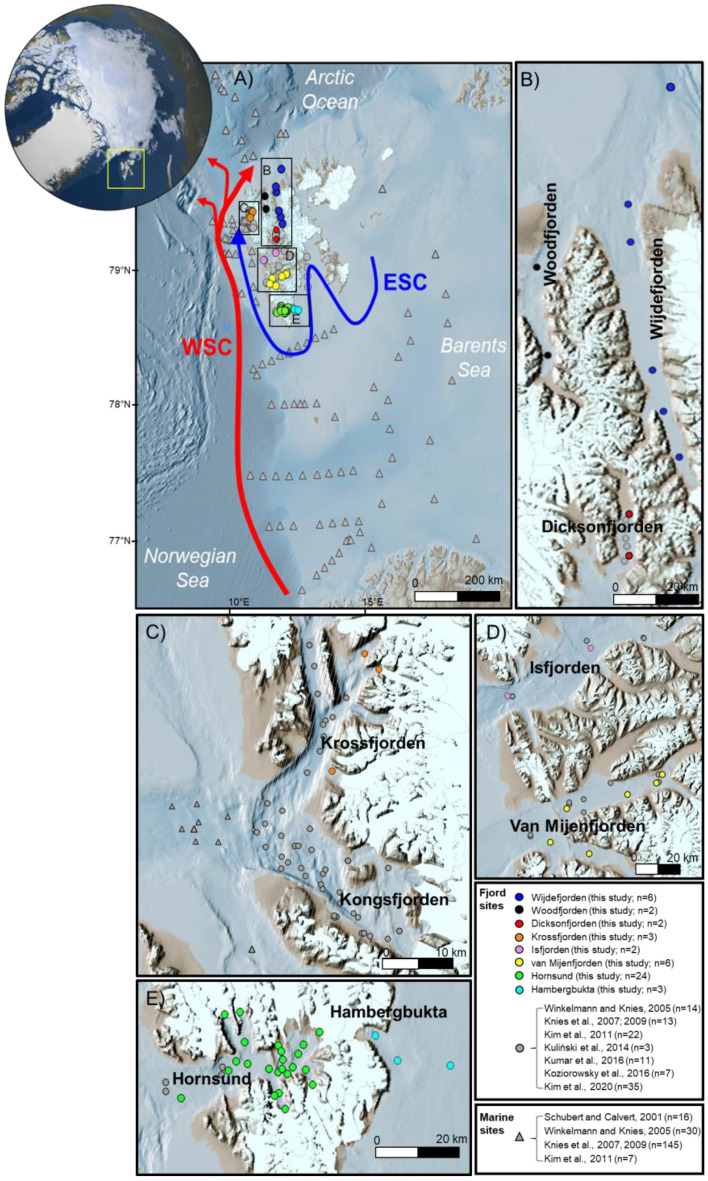
Map of the study area (yellow box) showing (**A**) the Svalbard archipelago and (**B**–**E**) the sampling sites considered in this study. White land areas represent the glacier coverage at present. ESC and WSC denote East Spitsbergen Current and West Spitsbergen Current, respectively. The map was generated using QGIS v3.14 (https://qgis.org/en/site/forusers/visualchangelog314/) based on IBCAOv4^70^ (https://www.ngdc.noaa.gov/mgg/bathymetry/arctic/). See also Supplementary Table S1 for detailed sample information.

The high-Arctic Svalbard archipelago (74–81° N, 10–35° E), with glaciers covering approximately 57% of its land masses, is located at the gateway between the North Atlantic and Arctic Oceans (Fig. 1). Spitsbergen is the largest island in the archipelago, characterized by its numerous fjords, some of which experience seasonal sea surface freezing during winter^8^. In recent decades, the fjords on the west coast of Spitsbergen have undergone significant hydrographic changes attributed to the intensified West Spitsbergen Current (WSC), which brings warm and saline Atlantic water into the fjords^9–11^. Furthermore, the Svalbard archipelago has experienced rising air temperatures and increased precipitation, leading to the retreat of marine- and land-terminating glaciers and increased freshwater discharge^12–15^. Due to increased WSC inflow and freshwater runoff, these hydrographic changes can potentially affect marine primary productivity and trigger the release of substantial amounts of terrestrial sediments to the fjords^8,13,16^. Consequently, these changes can alter the sources of sedimentary OC and significantly impact long-term OC burial in fjord sediments. Hence, the Spitsbergen fjords are regarded as areas susceptible to global warming^17,18^, making them ideal sites for studying the responses of high-Arctic fjords to current and projected future climate change.

Previous studies in Svalbard fjords have examined the spatial variability of OC composition in surface sediments and assessed the relative proportions of marine and terrestrial OC using bulk elemental (N_org_/TOC ratio) and isotopic (δ^13^C_org_) parameters^19–23^. However, these approaches often assume that the selected end-member (marine versus terrestrial) values represent the entire Svalbard region and treat all land-derived OC as a single terrestrial source. Nonetheless, it's important to recognize that land-derived OC is itself heterogeneous, comprising OC derived from both plants and soils (referred to as “terrestrial biogenic” OC), and bedrocks (referred to as “petrogenic” OC). Consequently, relying solely on conventional bulk organic parameters does not adequately capture the diverse sources of terrestrial OC in the sedimentary OC pool. Furthermore, only a limited number of studies have investigated surface sediments at the molecular level in Svalbard fjords, aiming to gain insights into different terrestrial OC sources^23–25^. Notably, the relative proportion of petrogenic and aged soil-derived OC to the total OC pool in Svalbard fjords remains largely unknown, despite their potential implications for active regional carbon cycling^24–26^. Therefore, a more comprehensive study investigating the contributions of petrogenic and soil-derived OC across Svalbard fjords is still necessary.

In this study, we conducted comprehensive analyses on surface sediments (n = 48) collected from eight Svalbard fjords (Fig. 1, see also Table S1 in Supplementary Information). Our analyses encompassed both bulk (stable and radioactive isotopic compositions) and molecular (lignin phenols) parameters to enhance our understanding of OC sources. Additionally, we performed grain size and Nd isotope analyses to identify geological characteristics related to the surrounding bedrock types. Furthermore, we analyzed bedrock (n = 17), soil (n = 28), and plant (n = 12) samples to constrain end-member values of OC sources (Supplementary Table S2 and Fig. S1). Our main objectives were to (1) constrain the diverse sources of sedimentary OC and (2) estimate their relative contributions to fjord sediments. The results of our study provide valuable qualitative and quantitative assessments of OC sources and compositions in the fjords of Svalbard. Furthermore, this study advances our understanding of carbon cycling in Svalbard fjords by offering new insights into biomarker data and highlighting the significant contribution of petrogenic and aged soil-derived OC to the present-day sedimentary OC pool.

## Results

### Sediment properties

The mean grain size of the fjord surface sediments was between 5.8 μm and 16.7 μm (average ± standard deviation (SD); 9.9 ± 3.2 μm, n = 48), and a predominant proportion of silt (63.7 ± 6.5%), followed by clay (28.1 ± 8.8%), and sand (8.2 ± 7.2%) was observed (Supplementary Table S1 and Fig. S2). The sorting value ranged from 1.4 to 2.4 (1.8 ± 0.2, n = 48), showing that most of the surface sediments in the fjords were poorly sorted (Supplementary Table S1 and Fig. S2).

### Bulk elemental and isotopic compositions

The total organic carbon (TOC) content in the fjord surface sediments varied between 0.12 wt.% and 2.80 wt.% (Supplementary Table S1 and Fig. S3). The total nitrogen (N_tot_) and total organic nitrogen (N_org_) contents ranged from 0.01 to 0.26 wt.% and 0.01 to 0.20 wt.%, respectively (Supplementary Table S1 and Fig. S3). The N_tot_/TOC ratios ranged from 0.04 to 0.20, whereas the N_org_/TOC ratios were lower, ranging from 0.01 to 0.13 (Supplementary Table S1 and Fig. S4). The δ^13^C_org_ values exhibited a wide range of variation between − 18.0‰ and − 26.9‰ (Supplementary Table S1 and Fig. S4), and the Δ^14^C_org_ values ranged from − 961.3‰ to − 219.0‰ (Supplementary Table S1).

In the plant samples collected around Longyearbyen, the TOC contents varied between 40.8 and 60.4 wt.% (47.7 ± 5.7 wt.%, n = 12; Supplementary Table S2). The soil samples collected around Longyearbyen and Ny-Ålesund showed the TOC contents ranging from 0.1 to 85.1 wt.% (9.9 ± 16.5 wt.%, n = 28), respectively. Most bedrock samples exhibited low TOC contents (0.3 ± 0.4 wt.%, n = 14), except for the coal samples (51.6 ± 7.0 wt.%, n = 3). The δ^13^C_org_ values of the plant samples ranged from − 35.2 to − 28.8‰ (− 31.4 ± 1.6‰, n = 12; Supplementary Table S2), whereas the soil samples displayed values of − 30.3‰ to − 17.5‰ (− 25.2 ± 2.8‰, n = 28). The δ^13^C_org_ values of the bedrock samples varied between − 29.0 and − 23.2‰ (− 25.8 ± 2.0‰, n = 17).

### Nd isotopic compositions

In this study, we newly measured neodymium isotopes from detrital components of surface sediments (n = 19) and compiled previous measurements (n = 29)^27^. The detrital ε_Nd_ values ranged from − 24.9 to − 8.9 (− 14.2 ± 2.9, n = 48; Supplementary Table S1). The highest detrital ε_Nd_ value was found in Hambergbukta at an average value of − 9.2 ± 0.3 (n = 3), while the lowest value was found in Wijdefjorden at an average value of − 15.2 ± 0.6 (n = 6). Notably, the detrital ε_Nd_ in Hornsund showed a wide range of values from − 24.9 to − 10.1 (− 14.9 ± 3.9, n = 24).

### Lignin phenol signatures

We reported the total lignin phenol concentration (λ) as the sum of eight lignin-derived monomeric phenols, including vanillyl (V; vanillin, acetovanillone, and vanillic acid), syringyl (S; syringaldehyde, acetosyringone, and syringic acid), and cinnamyl (C; p-coumaric acid and ferulic acid) units normalized to TOC. In the surface sediments of the fjords, λ ranged from 0.001 to 0.52 mg/g OC, with an average of 0.14 ± 0.15 mg/g OC (n = 48). The highest concentration was observed in Wijdefjorden (0.40 ± 0.08 mg/g OC, n = 6), while the lowest value was found in Hornsund (0.03 ± 0.02 mg/g OC, n = 24; Supplementary Table S3). The ratios of syringyl to vanillyl (S/V) and cinnamyl to vanillyl (C/V) varied between 0.05 and 0.62 (0.26 ± 0.13, n = 48) and 0.05 and 2.44 (0.46 ± 0.55, n = 48), respectively. The CuO oxidation also produced 3,5-dihydroxybenzoic acid (3,5-Bd) with concentrations ranging from 0.003 to 0.76 mg/g OC (0.15 ± 0.19 mg/g OC, n = 48), and the ratio of 3,5-Bd to vanillyl phenols (3.5-Bd/V) varied between 0.41 and 4.22 (1.81 ± 1.04, n = 48). The acid-to-aldehyde (Ad/Al) ratios of vanillyl (V) phenols ((Ad/Al)v) varied between 0.09 and 2.01 (0.81 ± 0.46, n = 48).

The plant samples exhibited λ values ranging from 6.19 to 62.19 mg/g OC (26.10 ± 20.50 mg/g OC, n = 12; Supplementary Table S2). On the other hand, the soil samples displayed a concentration ranging from 0.14 to 12.41 mg/g OC (4.44 ± 4.72 mg/g OC, n = 28). The S/V and C/V ratios of the plant samples ranged from 0.01 to 0.44 (0.19 ± 0.16, n = 12) and 0.19 to 3.32 (1.03 ± 1.02, n = 12), respectively. For the soil samples, the S/V and C/V ratios ranged from 0.03 to 0.71 (0.30 ± 0.15, n = 28) and 0.01 to 0.32 (0.07 ± 0.07, n = 28), respectively. The 3,5-Bd concentrations of the plant and soil samples varied between 0.36 and 1.86 mg/g OC (1.00 ± 0.49 mg/g OC, n = 12) and between 0.03 and 3.59 mg/g OC (1.04 ± 1.24 mg/g OC, n = 28), respectively. The 3,5-Bd/V ratio of the plant and soil samples had average values of 0.28 ± 0.12 (n = 12) and 0.34 ± 0.28 (n = 28), respectively. The (Ad/Al)v ratios of the plant and soil samples ranged from 0.23 to 0.46 (0.33 ± 0.09, n = 12) and from 0.27 to 5.16 (1.37 ± 1.47, n = 28), respectively. Notably, lignin phenols were not detected in most of the freshly unweathered bedrocks (n = 14) analyzed in this study (Supplementary Table S2). However, lignin phenols and 3,5-Bd were detected in the coal samples (n = 3) at low concentrations (λ: 0.01 ± 0.01 mg/g OC, 3,5-Bd: 0.01 ± 0.01 mg/g OC), interestingly higher than in some surface sediments in Hornsund. The S/V and C/V ratios of the coal samples ranged from 0.05 to 0.11 (0.07 ± 0.03, n = 3) and from 0.05 to 0.07 (0.06 ± 0.01, n = 3), respectively. The 3,5-Bd/V and (Ad/Al)v ratios were found to be 0.47 ± 0.13 (n = 3) and 0.49 ± 0.16 (n = 3), respectively.

## Discussion

### Characteristics of sedimentary organic matter

The TOC values obtained from the fjord surface sediments (1.5 ± 0.7 wt.%, n = 48; Supplementary Table S1) were within the range of those previously reported for Svalbard fjords (1.7 ± 1.0 wt.%, n = 64)^19,21–23,28,29^. Notably, no apparent correlation was observed between the TOC content and the mean grain size of the surface sediments, which predominantly consisted of silt and clay fractions (Supplementary Fig. S2). Furthermore, the TOC content showed no clear relationship with the sediment sorting (Ф), which indicated that the fjord surface sediments were poorly sorted (Supplementary Fig. S2). Hence, the sediment samples analyzed in this study represent typically fine-grained sediments deposited in glacimarine environments within Svalbard fjords^8,30,31^.

**Figure 2:**
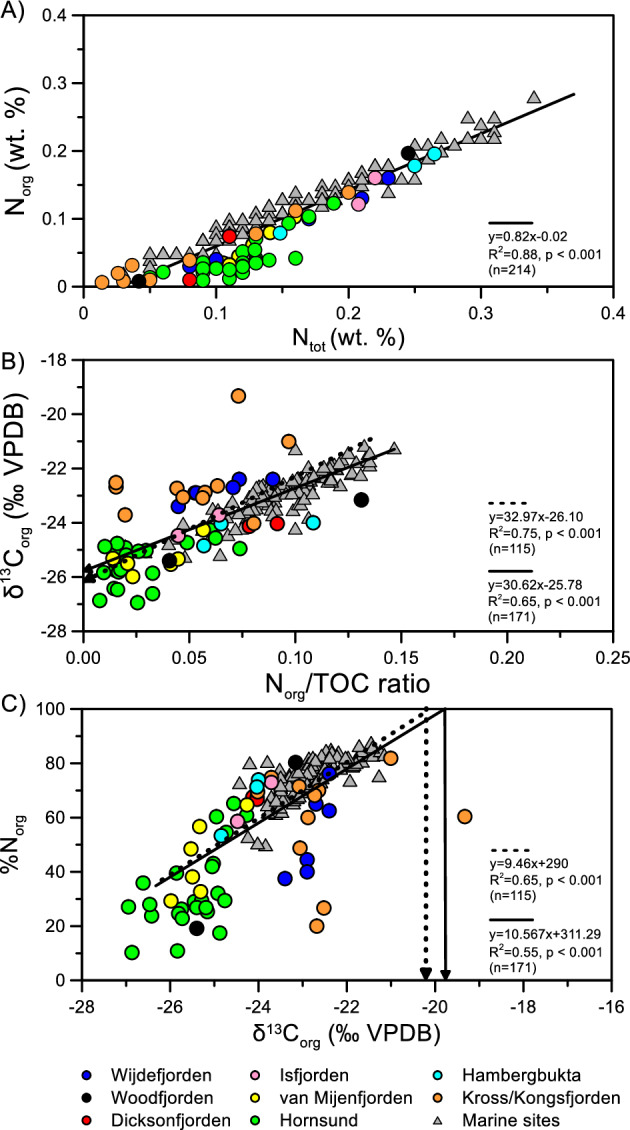
Scatter plots of (**A**) N_tot_ (wt.%) versus N_org_ (wt.%), (**B**) N_org_/TOC ratio versus δ^13^C_org_ (‰ VPDB), and (**C**) δ^13^C_org_ (‰ VPDB) versus %N_org_. Dotted lines indicate the relationships reported by Knies et al.^20^, while solid lines represent those obtained from this study.

Previous studies conducted in Svalbard fjords have revealed the presence of inorganic nitrogen (N) bound in clay minerals, which accounted for up to 70% of the N_tot_ content^19–21,28^_._ As depicted in the N_tot_ versus N_org_ plot (Fig. 2A), the surface sediments examined in this study also showed a significant contribution of inorganic N (45 ± 20%, n = 48). Therefore, it is strongly recommended to consider the fraction of N_inorg_ when assessing the source of organic matter in the Arctic region^18,32^. Consequently, the N_org_/TOC ratio, rather than the N_tot_/TOC ratio, serves as a better indicator for characterizing the sources of sedimentary OC (marine versus terrestrial) in Svalbard fjords. δ^13^C_org_ is another tool commonly used to identify sedimentary OC sources in Svalbard fjords^19,32^. The N_org_/TOC ratio versus δ^13^C_org_ plot suggests that the fjord surface sediments, as observed in previous studies of Svalbard fjords^19^, represent a mixture of terrestrial and marine OC (Fig. 2B, see also Supplementary Fig. S5). In general, C_3_ plant-derived terrestrial OC has depleted δ^13^C_org_ values between − 29.3 and − 25.5‰, while marine OC has more enriched δ^13^C_org_ values between − 17.0 and − 25.0‰^19,22,33–35^. Previous studies in Svalbard fjords have often used the similar end-member values for terrestrial and marine OC, as documented in Supplementary Table S5 and Fig. S6. However, it is noteworthy that in Kongsfjorden, where more research has been conducted, there is an overlap in the end-member values of terrestrial and marine OC. Consequently, assigning terrestrial and marine end-member values based on the conventional bulk indicator (δ^13^C_org_) to determine the quantitative proportions of sedimentary OC remains challenging.

**Figure 3:**
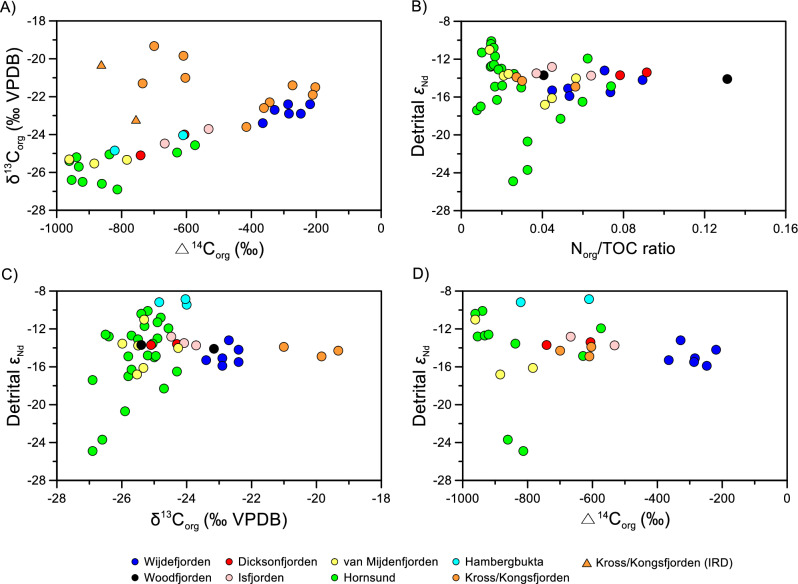
Scatter plots of (**A**) Δ^14^C_org_ (‰) versus δ^13^C_org_ (‰ VPDB), (**B**) Δ^14^C_org_ (‰) versus detrital ɛNd, (**C**) N_org_/TOC ratio versus detrital ɛNd, and (**D**) δ^13^C_org_ (‰ VPDB) versus detrital ɛNd. See also Supplementary Table S1 for detailed sample information.

Following the approach by Knies et al.^20^ and incorporating our new data, we attempted to assign terrestrial and marine OC end-member values based on the correlations between the N_org_/TOC ratio and δ^13^C_org_, as well as between δ^13^C_org_ and %N_org_ (defined as N_org_/N_tot_ %), respectively (Fig. 2). Assuming that N_org_ represents marine-originated N exclusively, a terrestrial end-member value of − 25.6‰ was obtained as the intercept at N_org_/TOC = 0, with a 95% confidence interval error range of − 25.8 to − 25.4‰ (Fig. 2B). Similarly, a marine end-member value of − 19.9‰ (− 20.1 to − 19.7‰ with a 95% confidence interval error range) was defined as 100% of %N_org_, i.e., 100% N_org_ in the N_tot_ fraction (Fig. 2C). These end-member values were slightly higher than those previously published (− 26.1‰ for the terrestrial end-member and − 20.1‰ for the marine end-member)^19^. This difference can be attributed to the larger variability in the data from Svalbard fjords, particularly for data from Kross/Kongsfjorden. It is worth noting that a previous study^29^ reported δ^13^C_org_ values ranging from − 23.8 to − 20.3‰ (− 22.6 ± 0.1‰, n = 4) for fine-grained ice-rafted debris (IRD) samples collected in Kross/Kongsfjorden. These δ^13^C_org_ values were relatively enriched compared to the soils collected around Kross/Kongsfjorden, which varied from − 27.1 to − 25.2‰ (− 26.1 ± 0.6‰, n = 15)^29^. Additionally, our newly acquired soil data from the Ny-Ålesund region exhibited similarly depleted δ^13^C_org_ values ranging from − 26.4 to − 17.5‰ (− 23.6 ± 2.5‰, n = 16; Supplementary Table S2), consistent with previously published soil data^27^. Notably, the δ^13^C_org_ values of the surface sediment samples collected near marine-terminating glaciers in Kross/Kongsfjorden fell within the range of the IRD samples (Fig. 3A, see also Supplementary Fig. S7). Furthermore, the surface sediment samples collected from Hornsund showed large variations in the N_org_/TOC ratio and δ^13^C_org_, which corresponded to the variations observed in detrital εNd (Fig. 3B,C). Detrital εNd is commonly used to identify changes in sediment provenance^27^ because bedrocks exhibit characteristic Nd isotopes that depend on their geological rock types and ages. These isotopes can be reflected in sedimentary εNd values without substantial fractionation during weathering and biological processes^36^. Compared to other fjords, the observed variability in εNd values in Hornsund primarily reflects the diverse bedrock geology in the surrounding catchment areas^27^, which ranges from Precambrian metamorphic rocks to Cenozoic sedimentary rocks^37^. Higher detrital εNd values were generally associated with sediments near the glacial outflow, which drains early Palaeozoic sedimentary rocks. In comparison, lower detrital εNd values were observed in innermost fjords dominated by Middle Jurassic to early Cretaceous sedimentary rocks^27,37^ (see Supplementary Figs. S8 and S9). This finding suggests that OC originating from some bedrocks can be transported into Hornsund, resulting in potentially large variations in the N_org_/TOC ratio and δ^13^C_org_. Consequently, our results indicate that bulk parameters such as the N_org_/TOC ratio and δ^13^C_org_ alone cannot fully resolve the heterogeneous sources of sedimentary OC in Svalbard fjords.

### Contribution of petrogenic OC

The Δ^14^C values of the surface sediments in Svalbard fjords were consistently depleted in ^14^C (− 666.9 ± 240.3‰, n = 28, Fig. 3A,D). This depletion aligns with findings from previous studies conducted in Hornsund and Kross/Kongsfjorden, where surface sediments exhibited Δ^14^C values ranging from − 952 to − 203‰^24,25,29^. Carbon dioxide (CO_2_) used for terrestrial or marine primary production, originating from the atmosphere, exhibits a positive ^14^C signal^38,39^, as demonstrated at Alert during the period of 2015–2019 with an average Δ^14^C value of 9.1 ± 6.4‰^40^. Therefore, fjord surface sediments that predominantly contain modern terrestrial or marine OC should not exhibit a strong depletion in ^14^C, assuming the absence of abiotic processes causing ^14^C offsets. As suggested by the previous study^29^, the significant depletion of ^14^C in the fjord surface sediments suggests that recently fixed terrestrial and marine OC alone is not the exclusive contributor to sedimentary OC. Instead, a substantial amount of old OC was also contributed, which could not be revealed solely by the N_org_/TOC ratio and δ^13^C_org_.

To identify the source of ^14^C-depleted OC in sedimentary OC in Svalbard fjords, we defined two OC components: ‘petrogenic’ OC, representing radiocarbon-‘dead’ OC sourced from bedrocks, and ‘biogenic’ OC, encompassing biomass fixed via photosynthesis in terrestrial and marine environments^7^. By assuming the end-member values of Δ^14^C_petrogenic_ =  − 1000‰ and Δ^14^C_biogenic_ = 9.1‰, we estimated the relative proportions of ‘^14^C-assessed’ petrogenic and biogenic OC to the sedimentary OC pool. The end-member Δ^14^C_org_ value of biogenic OC was assumed to be represented by the ^14^C composition of atmospheric CO_2_ at Alert during the period of 2015–2019^40^, taking into account the sampling period of our surface sediments (Supplementary Table S2). The petrogenic OC fraction of TOC varied between 22.6 and 96.2% (60.0 ± 23.8%, n = 28), while the biogenic OC fraction varied between 3.8 and 77.4% (33.0 ± 23.0%, n = 28). Among the studied fjords, van Mijenfjorden (87.7 ± 8.8%, n = 3) and Hornsund (84.4 ± 13.6%, n = 10) displayed higher relative proportions of petrogenic OC, whereas Wijdefjorden (70.5 ± 5.2%, n = 6) exhibited higher relative proportions of biogenic OC. These results indicate a substantial contribution of petrogenic OC to Svalbard fjords, consistent with previous findings in Kross/Kongsfjorden^29^. However, characterizing biogenic OC with a Δ^14^C of 9.1‰ alone is likely an oversimplification in Svalbard fjords. This is because biogenic OC encompasses not only recently fixed terrestrial and marine biomass but also pre-aged OC derived from soils, which can be transported to fjords through glacial erosion and/or meltwater discharge.

### Contribution of aged soil-derived OC

To enhance our understanding of the source of ^14^C-depleted OC in Svalbard fjords, we further defined biogenic OC comprising both more recently fixed terrestrial and marine biomass as well as pre-aged OC derived from soils. Previous studies conducted in Kross/Kongsfjorden indicated a low contribution of fresh plant- and soil-derived OC to the sedimentary OC pool, likely due to the limited soil formation in the cold Arctic environment^29,41^. However, a more recent study in the Ny-Ålesund region revealed potential inputs of deep active layer/permafrost OC in the Bayelva River and its river mouth^24^. Thus, considering the potential input of OC derived from pre-aged soils, we calculated the relative proportions of petrogenic and biogenic OC to sedimentary OC by applying the binary mixing model proposed by Galy et al.^42^ as follows:

1 $$ {\text{TOC}}_{{{\text{sample}}}} \times {\text{ Fm}}_{{{\text{sample}}}} = {\text{ TOC}}_{{{\text{sample}}}} \times {\text{ Fm}}_{{{\text{bio}}}} {-}{\text{ OC}}_{{{\text{petro}}}} \times {\text{ Fm}}_{{{\text{bio}}}} $$

where TOC_sample_ represents the TOC content (wt.%) in the sample, Fm_sample_ is the measured ^14^C composition of the sample expressed as the fraction of modern C, Fm_bio_ is the ^14^C composition of biogenic OC, and OC_petro_ is the content of petrogenic OC in wt.%. Although this approach was successfully applied to the Beaufort Sea^43^, we did not observe a similar linear relationship between the TOC_sample_ and TOC_sample_ × Fm_sample_ in Svalbard fjords (Supplementary Fig. S10). This discrepancy can be attributed to the fact that the assumption of a constant background level of petrogenic OC in all investigated samples, as suggested by Galy et al.^42^, is not applicable in Svalbard fjords. This might be due to the presence of complex bedrock types in the catchment areas (Supplementary Fig. S8)^27,37^, as well as the varying rates of glacier retreat observed in Svalbard fjords^44^. Consequently, different quantities of terrestrial sediments, including terrestrial OC, would have been supplied to the fjords.

### Four OC source apportionments

**Figure 4:**
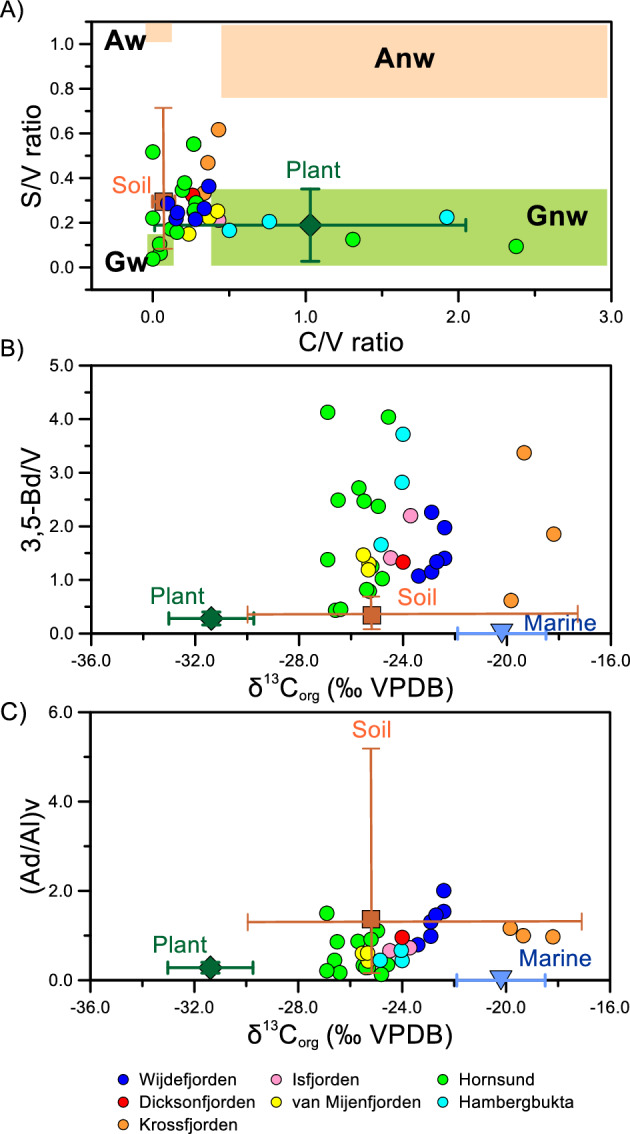
Scatter plots of the (**A**) C/V ratio versus S/V ratio, (**B**) δ^13^C_org_ (‰ VPDB) versus 3,5-Bd/V, and (**C**) δ^13^C_org_ (‰ VPDB) versus (Ad/Al)v. The range of different vascular plant tissues^43,71^ is also shown in the plot (*Aw* angiosperm woody, *Anw* angiosperm non-woody, *Gw* gymnosperm woody, *Gnw* gymnosperm non-woody). Note that no samples from the Woodfjorden were available for the lignin phenol analyses.

To further explore the potential contribution of aged soil-derived OC to Svalbard fjords, we examined lignin phenols obtained through alkaline CuO oxidation. Lignin phenols are widely used as valuable terrestrial biomarkers due to their unique synthesis by higher vascular plants^45,46^. The ratios of S/V and C/V, which are indicators of lignin sources, have been utilized to assess the relative proportions of non-woody angiosperm to woody gymnosperm contributions in various aquatic environments^46,47^. In the surface sediments of the fjords, lignin phenols mainly consisted of a mixture of non-woody gymnosperm OC with inputs from gymnosperm wood-derived tissues. The fjord surface sediments exhibited similar lignin phenol characteristics to the plants and soils collected around Ny-Ålesund and Longyearbyen (Fig. 4A). However, the ratio of 3,5-Bd/vanillyl phenols (3,5-Bd/V), an indicator of the degradation state of complex terrestrial organic mixtures^48^, and used as a proxy for the relative contributions of soil-derived OC versus vascular plant-derived OC in aquatic environments^49^, was generally higher in the fjord surface sediments compared to the plants and soils (Fig. 4B). The ratio of vanillic acid to vanillin ((Ad/Al)v) is commonly used as an index for lignin oxidative degradation by aerobic degraders^50–52^. Ratios of (Ad/Al)v lower than 0.3 are commonly associated with relatively fresh vascular plant detritus, whereas ratios exceeding 0.5 are typically observed in extensively altered soils with significantly depleted Δ^14^C_org_ signatures^45^. Many of the surface sediments in Svalbard fjords exhibited (Ad/Al)v ratios well above 0.5 (Fig. 4C). Furthermore, principal component analysis (PCA) based on the concentrations of lignin phenols and 3,5-Bd also revealed that most of the fjord surface sediments were distinct from the investigated plants and soils (Supplementary Fig. S11). These findings suggest that the terrestrial OC in the fjord surface sediments had undergone more significant degradation compared to the plants and soils analyzed in this study.

In addition to exposed soils resulting from glacier retreat, another potential source of aged and highly degraded soil-derived OC is subglacial sediments in glacier-covered areas, such as the Svalbard catchments. During glacier advances, plants, soils, and fjord sediments were overridden by glaciers, leading to the burial of substantial amounts of OC within subglacial sediments^53–55^. As a result of climate warming, melting glaciers can transport these subglacial sediments and associated OC to the fjords^1^, contributing to the presence of aged and highly degraded soil-derived OC. It is worth noting that the soil samples analyzed in this study were obtained from the active layers in glacier-exposed areas. Interestingly, no significant differences were observed between samples collected at deeper depths (20–30 cm) of the soil profiles and those collected at shallower depths (0–10 cm) in the vicinity of the retreating land-terminating glacier in the Ny-Ålesund region (Supplementary Fig. S11). Therefore, we did not differentiate between OC derived from active layer/permafrost soils and subglacial sediments but instead collectively referred to them as ‘aged soil-derived’ OC for further discussion. Overall, our findings suggest the presence of highly degraded and aged soil-derived OC in the fjord surface sediments, likely originating from subglacial sediments and/or active layer/permafrost soils. These findings highlight the importance of considering this source when studying OC dynamics in Svalbard fjords.

**Figure 5:**
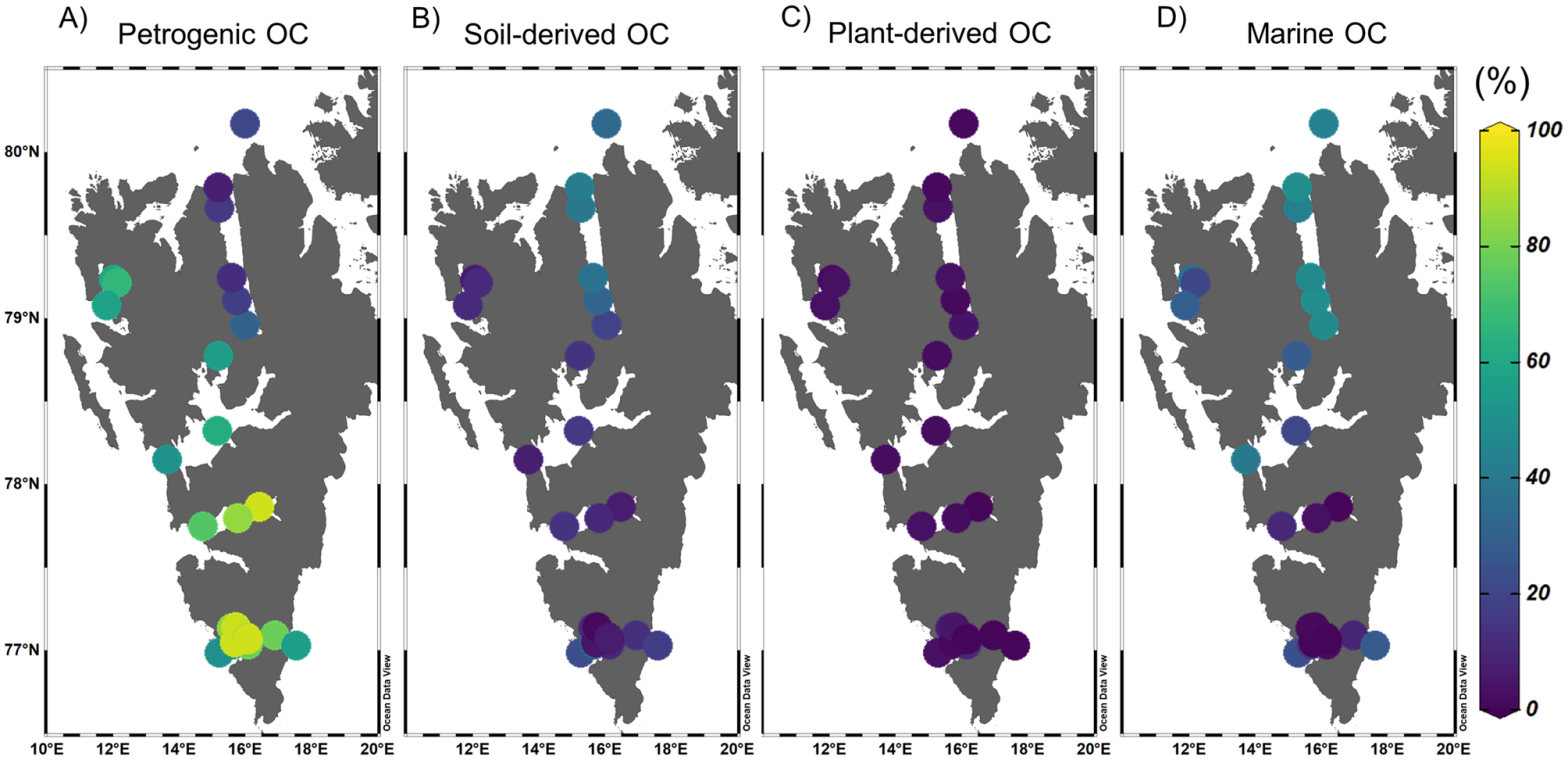
Spatial distribution of (**A**) petrogenic OC (%), (**B**) Soil-derived OC (%), (**C**) Plant-derived OC (%), and (**D**) marine OC (%) calculated based on Δ^14^C_org_ (‰), δ^13^C_org_ (‰ VPDB), and lignin phenols. The map was generated with the Ocean Data View version 5.6.3 (GlobalHR, https://odv.awi.de).

To estimate the relative proportions of petrogenic, soil-derived, plant-derived, and marine OC in the sedimentary OC of Svalbard fjords, we utilized a four-source apportionment approach based on Δ^14^C_org_, δ^13^C_org_, and (Ad/Al)v ratio, employing a Monte Carlo (MC) analysis^56,57^. In the initial stage, we constrained the end-member values for each OC source (Supplementary Table S6) using newly generated and compiled data (see Supplementary Table S2). The proportions of OC sources varied, with petrogenic OC comprising the highest proportion at 59.0 ± 28.1% (n = 25), followed by marine OC at 21.8 ± 18.5% (n = 25), soil-derived OC at 16.8 ± 12.1% (n = 25), and plant-derived OC at 2.5 ± 2.2% (n = 25) (Fig. 5, see also Supplementary Fig. S12). The relative proportions of petrogenic OC were found to be higher in van Mijenfjorden (84.2 ± 10.1%, n = 3) and Hornsund (79.2 ± 18.3%, n = 8), possibly due to their higher glacier retreat rates compared to other fjords^44^. Notably, the surface sediments in Wijdefjorden exhibited more enriched δ^13^C_org_ values than the other fjords, resulting in relatively higher contributions of marine-derived OC (46.8 ± 2.8%, n = 6) and soil-derived OC (33.9 ± 8.3%, n = 6) to the sedimentary OC pool. Our study demonstrated that the four-source apportionment approach based on Δ^14^C_org_, δ^13^C_org_, and the (Ad/Al)v ratio can provide reasonable estimates of the relative contributions of different OC sources to sedimentary OC in Svalbard fjords. However, further investigation is necessary to address the issue of overlapping end-member values of δ^13^C_org_, particularly in Kross/Kongsfjorden, by analyzing additional background samples such as soils, subglacial sediments, and fjord sediments collected in the vicinity of tidewater glacier fronts. This further analysis would help in better constraining the end-member values for each parameter considered and improving the accuracy of the apportionment approach.

### Potential future implication on fjord carbon dynamics

In temperate fjords with vegetation-dominated catchments, such as New Zealand fjords, the input of petrogenic OC is negligible^7^. In such environments, the effective burial of OC derived from more recently fixed terrestrial and marine biomass in fjord sediments can remove OC from the active carbon cycle. However, high-latitude fjords with glacier-dominated catchments, like those in Svalbard, experience the export of previously sequestered petrogenic OC to complex fjord systems^58^. This old OC can be remineralized during transport or within the fjords, thus impacting the active carbon cycle as a source of CO_2_ to the atmosphere^59,60^. In the case of the Svalbard fjords, the marine-terminating glaciers have undergone rapid retreat in recent decades^61^. As a result, there has been an increased export of OC from the hinterland to the fjords^23,62^, which is expected to accelerate in the near future due to projected climate warming^6^. Consequently, the amplified input of petrogenic and aged soil OC to Svalbard fjords may contribute to atmospheric CO_2_, despite its refractory nature, if this old OC is not effectively reburied but instead undergoes remineralization^59,60,63^. This positive carbon feedback mechanism in Svalbard fjords requires further investigation.

## Conclusions

In this study, we conducted a comprehensive analysis of the OC sources in surface sediments from Svalbard fjords, employing a combination of bulk and molecular geochemical parameters. Our findings revealed that the surface sediments in Svalbard fjords exhibit depleted Δ^14^C_org_ values and a wide range of N_org_/TOC and δ^13^C_org_ values. These observations suggest that the sedimentary OC sources in Svalbard fjords cannot be adequately explained by a simple binary mixing model considering only marine and terrestrial OC. To overcome this limitation, we applied a MC approach, utilizing Δ^14^C_org_, δ^13^C_org_, and (Ad/Al)v values, to estimate the relative contributions of different OC sources to the sedimentary OC pool. This approach allowed us to discern the proportions of petrogenic, soil-derived, plant-derived, and marine OC in the sediment samples. Our analysis provides important insights into the sources of OC in Svalbard fjords, with a particular emphasis on the substantial contributions of petrogenic and aged soil-derived OC to the recent sedimentary OC pool. Furthermore, our study highlights the potential implications of predicted climate warming on the active carbon cycle in the Svalbard fjords. With the anticipated acceleration of glacier retreat, there will be an increased export of petrogenic and aged soil-derived OC to these fjords. This influx of OC has the potential to release additional CO_2_ into the atmosphere, thereby influencing the dynamics of the active carbon cycle^25^. Furthermore, the increased influxes of land-derived OC could alter ecosystem structure and function in Svalbard fjords, as demonstrated in Greenland fjords^64^.

## Materials and methods

### Sampling of marine surface sediments, plants, and soils

We collected a total of 45 surface sediment samples using a giant box corer (50 × 50 × 50 cm^3^) from seven Svalbard fjord systems: Wijdefjorden (n = 6), Woodfjorden (n = 2), Dicksonfjorden (n = 2), Isfjorden (n = 2), van Mijenfjorden (n = 6), Hornsund (n = 24), and Hambergbukta (n = 3). These samples were obtained during Korea-Norway International joint cruises in Svalbard conducted in 2015, 2016, 2017, and 2019 aboard *R/V Helmer Hanssesn* of UiT The Arctic University of Norway (Fig. 1, see also Supplementary Table S1). Additionally, three surface sediment samples were collected using a multicorer in Krossfjorden in 2015 on *MS Teisten*. In July 2021, we sampled the most dominant vascular plant species (*Cassiope, Salix*, Moss, and *Dryas*) and the 5–10 cm depth of soils (n = 4) with three replicates each in Longyearbyen (see Supplementary Table S2 and Fig. S1). Additionally, we utilized soil samples collected around Ny-Ålesund at depths of 0–10 cm (n = 12) and 20–30 cm (n = 4), previously used by Kim et al.^65^ and Jung et al.^66^. All plant samples used in this study were collected with the permission of the governor of Svalbard (RiS-ID 10547), and the plant species were identified by Yoo Kyung Lee from the Korea Polar Research Institute (KOPRI). These materials are deposited at KOPRI. Furthermore, in addition to the 14 bedrock samples used by Jang et al.^27^, we collected three coal samples near Ny-Ålesund in 2022. Please refer to Supplementary Table S2 for more details on the sample locations and characteristics.

### Grain size analysis

The grain size distributions were determined following a procedure previously reported by Kim et al.^43^. Briefly, samples (~ 1 g) oxidized with 5 mL of H_2_O_2_ (35%) were analyzed for grain sizes smaller than 63 μm using a Mastersizer 3000 laser particle size analyzer (Malvern Panalytical B.V, Netherlands) at KOPRI. The analytical precision was as follows: D(10): 37.5 ± 0.3 μm, D(50): 71.4 ± 0.2 μm, and D(90): 104.0 ± 0.0 μm. Mean grain size and sediment sorting were estimated according to Folk and Ward^67^.

### Bulk organic geochemical analysis

Bulk elemental and stable isotope analyses were conducted following the methods described by Kim et al.^43^. In brief, the TOC content of samples decalcified with 10% HCl for 24 h, the nitrogen contents in both bulk (total nitrogen, N_tot_) and KOBr/KOH-treated (inorganic nitrogen, N_inorg_) samples, and carbon isotopic compositions were determined at KOPRI using an elemental analyzer (Thermo Electron Corporation Flash EA 2000, Thermo Fisher Scientific, Germany) coupled with an isotope ratio mass spectrometer (Finnigan Delta Plus, Thermo Fisher Scientific, Germany). The carbon isotope ratios of TOC (δ^13^C_org_) were reported using δ notation (per mil) with respect to the Vienna Pee Dee Belemnite (VPDB). The analytical precision was better than 0.5 wt.% and 0.5‰ for carbon and 0.5 wt.% for nitrogen. Accelerator mass spectrometry (AMS) radiocarbon (^14^C) analyses of TOC were performed at the MICADAS radiocarbon laboratory at the Alfred-Wegener Institute (AWI, Bremerhaven, Germany) and the Center for Applied Isotope Studies at the University of Georgia (CAIS, Georgia, USA), following standard procedures. Radiocarbon results were presented in *Delta* notation (Δ^14^C_org_, ‰), as defined by Stuiver and Polach^68^.

### Neodymium isotope analysis

Sequential extraction from bulk sediments and Nd isotopic analysis of the remaining detrital fractions were conducted according to the procedure described by Jang et al.^27^. Briefly, sediments (~ 100 mg) were chemically leached to obtain detrital fractions by removing the carbonate and authigenic fractions. All detrital fraction analyses were performed using a thermal ionization mass spectrometer (TIMS, Triton, Thermo Scientific) at KOPRI. The Nd isotope ratios were normalized to a ^144^Nd/^146^Nd value of 0.7219 to correct for mass fractionation. The analytical precision was 0.3 ε_Nd_ units for the measured Nd isotopic compositions.

### Lignin phenols analysis

CuO oxidation of the samples was and analyses were conducted following the method described by Kim et al.^43^. Briefly, microwave-assisted alkaline CuO oxidation was carried out on ~ 400 mg of the samples using a Microwave Digestion System (MARS 6 microwave, CEM Corporation, USA) at 150 °C for 1.5 h. All analyses were performed at KOPRI using an Agilent 7890B GC coupled to a 5977B Series Mass Selective Detector (Agilent Technologies, Santa Clara, CA, USA) operating in single ion monitoring (SIM) mode using a DB1-MS capillary column (30 m × 0.25 mm, 0.25 μm, Agilent J&W). The analytical precision associated with the lignin phenol concentrations was typically less than 10%.

### Statistical analysis

PCA was performed to provide an overview of the distribution of lignin phenols in fjord surface sediments, plants, and soils. When some components fell below the detection limit, a value of one-half of the minimum value detected for that variable across the entire dataset was assigned as the limit of detection^69^. The PCA of the lignin phenol concentrations was performed using R software version 4.0.3, specifically utilizing the FactoMineR package. To estimate the relative contributions of petrogenic, soil-derived, plant-derived, and marine OC to sedimentary OC in the fjords, a mixing model based on a Monte Carlo (MC) approach was employed (see Supplementary Information). This model incorporated Δ^14^C_org_, δ^13^C_org_, and (Ad/Al)v values (see Supplementary Table S6) and accounted for natural variations in end-members as well as measurement uncertainties, following previous studies^56,57^. In this statistical model, the variability of each end-member (i.e., petrogenic, soil-derived, plant-derived, and marine OC sources) was described as normal distributions with mean and standard deviations. The MC analysis was performed using R software version 4.0.3, with the MixSIAR package.

### Ethical approval

The authors promise that the experimental research and field studies on plants complied with relevant institutional, national, and international guidelines and legislation.

### Supplementary Information

Supplementary Information.

## Data Availability

All data generated and compiled in this study are presented in the Supplementary Information and will be available from the corresponding author upon request.
