# Supplementary material for: Large contributions of petrogenic and aged soil-derived organic carbon to Arctic fjord sediments in Svalbard
Source: Sci Rep. 2023 Oct 20;13:17935. doi: 10.1038/s41598-023-45141-z (PMC10589343; doi:10.1038/s41598-023-45141-z)
Supplement: Supplementary file 1 — Supplementary Information. [file 41598_2023_45141_MOESM1_ESM.docx]

**Supplementary Information**

**Large contributions of petrogenic and aged soil-derived organic carbon to Arctic fjord sediments in Svalbard**

Dahae Kim^1,2^, Jung-Hyun Kim^1,^*, Youngkyu Ahn^1,3^, Kwangchul Jang^1^, Ji Young Jung^1^, Minji Bae^1,3^, Seung-Il Nam^1^

^1^Korea Polar Research Institute, 26 Songdomirae-ro, Yeonsu-gu, Incheon 21990, South Korea

^2^Department of Marine Science and Convergence Technology, Hanyang University ERICA campus, 55 Hanyangdaehak-ro, Sangnok-gu, Ansan-si, Gyeonggi-do 15588, South Korea

^3^Department of Marine Science, Inha University, 100 Inha-ro, Michuhol-gu, Incheon 22212, South Korea

***Correspondence:**
Jung-Hyun Kim
jhkim123@kopri.re.kr

**Supplementary Methods**

**Monte Carlo (MC) approach**

In this study, we defined four distinct OC sources: petrogenic, soil-derived, plant-derived, and marine OC. To differentiate sedimentary OC sources, we applied dual-carbon (Δ^14^C_org_ and δ^13^C_org_) isotope and lignin parameter ((Ad/Al)v) measurements, utilizing a mass balance mixing model. The relative contributions of each source to surface sedimentary OC were estimated using a Monte Carlo (MC) approach, following established methods in previous studies^[1-2]^. For Svalbard fjords, we obtained end-member values for petrogenic, soil-derived, plant-derived, and marine OC, as detailed below and summarized in Table S6. In this approach, random sampling was performed from the normal distributions, ensuring a mass balance based on the measurement errors of the standard deviations. Solutions were generated from 100,000 random samplings of probability density functions. The MC calculations were executed using R software version 4.0.3, with the MixSIAR package.

**Defining end-member values for four sources**

***Petrogenic OC:***

We established end-member values for petrogenic OC by analyzing 14 bedrock samples previously studied by Jang et al.^[3]^. Additionally, we analyzed three coal samples from the Ny-Ålesund area for this study (Supplementary Table S2). We calculated average values and standard deviations (SD) for δ^13^C_org_ and (Ad/Al)v. The average values for the petrogenic source (Supplementary Table S6) were approximately -25.74±1.89‰ (n=18) for δ^13^C_org_ and 0.49±0.16 (n=18) for (Ad/Al)v. For the Δ^14^C_org_ value, we adopted the reported value for the coal sample (-1000.0‰) from Kim et al.^[4]^, with an assumed SD of 0, which served as the end-member value for petrogenic OC.

***Soil-derived OC:***

To establish end-member values for soil-derived OC, we conducted analyses of δ^13^C_org_ and (Ad/Al)v using 28 soil samples collected from the areas around Longyearbyen and Ny-Ålesund (Supplementary Table S2). Additionally, we compiled previously published data on δ^13^C_org_ and Δ^14^C_org_ from Kim et al.^[4]^ (n=15) and Kusch et al.^[5]^ (n=3) (Supplementary Table S2). The soil samples considered in this study encompassed a range of depths, from shallow (0-5 cm) to deeper (60-85 cm). We calculated average values and standard deviations from the newly generated and compiled δ^13^C_org_, (Ad/Al)v, and Δ^14^C_org_ data (Supplementary Table S6). The soil samples exhibited a wide range of δ^13^C_org_ values, ranging from −30.20‰ to −17.50‰ (with an average of −25.48±2.25‰, n=46), (Ad/Al)v values ranging from 0.01 to 5.16 (with an average of 1.37±1.47, n=28), and Δ^14^C_org_ values ranging from −960.8‰ to −107.1‰ (with an average of −344.4±358.9‰, n=18). It should be noted that defining soil end-member values in the Svalbard region poses challenges due to limited data availability. While we attempted to select soil samples that did not contain coals based on visual inspection, we acknowledge that we cannot fully exclude the possibility of petrogenic contributions in some samples, at least for now. Furthermore, our soil data are skewed toward shallow surface soils. This is partly due to slow soil development in the region, resulting in predominantly shallow soil depths across most of Svalbard. Additionally, we could not differentiate between shallow and deeper soil OC sources due to inadequate data. Indeed, deeper active layer samples (beyond 30 cm depth) contained more ^14^C-depleted OC^[5]^. Given the heterogeneity of the soils, we recognize the need for more extensive soil sampling in the future to refine these end-member values and provide a more comprehensive understanding of soil-derived OC sources in the Svalbard region.

***Plant-derived OC:***

To establish end-member values for plant-derived OC, we analyzed 12 plant samples collected around Longyearbyen for δ^13^C_org_ and (Ad/Al)v (Supplementary Table S2). From these analyses, we derived average values of −31.38±1.63‰ (n=12) for δ^13^C_org_ and 0.33±0.09 (n=12) for (Ad/Al)v (Supplementary Table S6). It is important to note that atmospheric CO_2_ used for terrestrial primary production exhibits a positive ^14^C signal^[6-7]^. Therefore, we used the average value of 9.1±6.4‰ (n=48) from the Alert station (Canadian high Arctic) during the period of 2015-2019^[8]^ as the end-member Δ^14^C_org_ value for plant-derived OC (Supplementary Table S6). This assumption considers it to be representative of the ^14^C composition of atmospheric CO_2_ during the sampling period of our surface sediments.

***Marine OC:***

As lignin phenols are exclusively produced by land vascular plants, we assigned a (Ad/Al)v value of 0±0 to marine OC (Supplementary Table S6). For δ^13^C_org_, we adopted the marine end-member value from the particulate organic carbon (POC) data reported in Isfjorden, Kongsfjorden, and Hornsund (−22.5±1.8‰, n=15)^[9]^ and Kongsfjorden (−19.0‰, n=5)^[10]^. The average δ^13^C_org_ value of −20.8±2.5‰ (n=20), closely aligning with the value of −19.9±0.2‰ determined using the approach by Knies et al.^[11]^ (see Fig. 2), was assigned as the marine end-member value (Supplementary Table S6). It is important to note that, similar to terrestrial primary production, CO_2_ utilized for marine primary production, sourced from the atmosphere, typically exhibits a positive ^14^C signal^[6-8]^. Modelled DIC data in Hornsund ranged from 0.5 to 106.9‰ in Δ^14^C_org_^[12]^. For C16:0 alkanoic acid in fjord sediments in Kongsfjorden, Δ^14^C_org_ values were 5.3±4.9‰ (n=2)^[5]^. Additionally, POC data reported in the northwestern part of the Svalbard Archipelago exhibited a positive Δ^14^C_org_ value of 5.2±6.3 (n=21)^[13]^ (Supplementary Table S2). Taking these factors, we adopted a marine OC end-member value of Δ^14^C_org_ to be 5.2±6.3‰ (Supplementary Table S6), which was obtained from the POC measurements around the Svalbard Archipelago.

Table S1. Sample information and data obtained from fjord surface sediments in this study. Note that “a” and “b” indicate the data published by Kim et al.^[14]^ and Jang et al.^[3]^, respectively. NA denotes “not available”.

| Sample name | Location | Latitude | Longitude | Sampling date  (mm/yyyy) | Sample type | Sample depth  (cm) | Water depth  (m) | TOC (wt. %) | N_tot_ (wt. %) | N_org_ (wt. %) | δ^13^C_org_  (‰ VPDB) | Δ^14^C_org_ (‰) | Detrital  εNd | Mean grain  size (um) | Sorting (Ф) |
| --- | --- | --- | --- | --- | --- | --- | --- | --- | --- | --- | --- | --- | --- | --- | --- |
| HH17-1107 | Wijdefjorden | 79.000 | 16.220 | 07/2017 | surface sediment | 0-1 | 158 | 0.67^a^ | 0.08^a^ | 0.03 | -23.4^a^ | -364.9 | -15.3^b^ | 11.46 | 1.72 |
| HH17-1108 | Wijdefjorden | 79.160 | 15.960 | 07/2017 | surface sediment | 0-1 | 145 | 0.76^a^ | 0.10^a^ | 0.04 | -22.9^a^ | -284.2 | -15.1^b^ | 8.99 | 1.47 |
| HH17-1109 | Wijdefjorden | 79.300 | 15.780 | 07/2017 | surface sediment | 0-1 | 112 | 0.75^a^ | 0.10^a^ | 0.04 | -22.9^a^ | -247.7 | -15.9^b^ | 9.50 | 1.57 |
| HH17-1110 | Wijdefjorden | 79.740 | 15.420 | 07/2017 | surface sediment | 0-1 | 148 | 1.36^a^ | 0.17^a^ | 0.10 | -22.4^a^ | -286.9 | -15.5^b^ | 15.49 | 1.65 |
| HH17-1111 | Wijdefjorden | 79.870 | 15.380 | 07/2017 | surface sediment | 0-1 | 163 | 1.79^a^ | 0.23^a^ | 0.16 | -22.4^a^ | -219.0 | -14.2^b^ | 16.67 | 1.84 |
| HH17-1086 | Wijdefjorden | 80.270 | 16.210 | 07/2017 | surface sediment | 0-1 | 322 | 1.84^a^ | 0.21^a^ | 0.13 | -22.7^a^ | -328.3 | -13.2^b^ | 15.21 | 1.92 |
| HH12-964GC | Woodfjorden | 79.650 | 13.750 | 07/2012 | surface sediment | 0-1 | 173 | 1.50 | 0.25 | 0.20 | -23.2 | NA | -14.1^b^ | NA | NA |
| HH12-969GC | Woodfjorden | 79.350 | 13.960 | 07/2012 | surface sediment | 0-1 | 50 | 0.20 | 0.04 | 0.01 | -25.4 | NA | -13.7^b^ | NA | NA |
| HH19 BOX 847 | Isfjorden | 78.334 | 15.331 | 07/2019 | surface sediment | 0-1 | 257 | 2.72 | 0.21 | 0.12 | -24.5 | -667.2 | -12.8 | 8.29 | 1.63 |
| HH19 BOX 850 | Isfjorden | 78.153 | 13.741 | 07/2019 | surface sediment | 0-1 | 425 | 2.51 | 0.22 | 0.16 | -23.7 | -531.9 | -13.7 | 11.54 | 1.74 |
| HH19 BOX 855 | van Mijenfjorden | 77.856 | 16.658 | 07/2019 | surface sediment | 0-1 | 33 | 2.48 | 0.11 | 0.03 | -25.3 | -961.3 | -11.0 | 6.84 | 1.59 |
| HH19 BOX 858 | van Mijenfjorden | 77.826 | 16.548 | 07/2019 | surface sediment | 0-1 | 68 | 2.14 | 0.12 | 0.04 | -25.5 | NA | -13.8 | 6.82 | 1.52 |
| HH19 BOX 863 | van Mijenfjorden | 77.786 | 16.006 | 07/2019 | surface sediment | 0-1 | 78 | 1.50 | 0.13 | 0.06 | -25.5 | -884.2 | -16.8 | 8.24 | 1.43 |
| HH19 BOX 868 | van Mijenfjorden | 77.734 | 14.873 | 07/2019 | surface sediment | 0-1 | 107 | 1.79 | 0.14 | 0.08 | -25.3 | -783.6 | -16.1 | 12.84 | 2.37 |
| HH19 BOX 872 | van Mijenfjorden | 77.567 | 15.287 | 07/2019 | surface sediment | 0-1 | 98 | 1.39 | 0.11 | 0.03 | -26.0 | NA | -13.6 | 5.75 | 1.55 |
| HH19 BOX 876 | van Mijenfjorden | 77.609 | 14.579 | 07/2019 | surface sediment | 0-1 | 128 | 1.82 | 0.16 | 0.10 | -24.3 | NA | -14.0 | 10.19 | 1.48 |
| HH19 BOX 895 | Hambergbukta | 77.045 | 17.163 | 07/2019 | surface sediment | 0-1 | 101 | 1.39 | 0.15 | 0.08 | -24.8 | -821.2 | -9.2 | 8.97 | 1.93 |
| HH19 BOX 898 | Hambergbukta | 76.997 | 17.354 | 07/2019 | surface sediment | 0-1 | 114 | 1.80 | 0.26 | 0.20 | -24.0 | NA | -9.4 | 15.98 | 1.84 |
| HH19 BOX 900 | Hambergbukta | 76.982 | 17.829 | 07/2019 | surface sediment | 0-1 | 126 | 2.74 | 0.25 | 0.18 | -24.0 | -610.6 | -8.9 | 9.67 | 2.03 |
| HH19 BOX 885 | Hornsund | 76.937 | 15.390 | 07/2019 | surface sediment | 0-1 | 161 | 1.97 | 0.19 | 0.12 | -24.6 | -573.7 | -11.9 | 10.95 | 1.79 |
| HH19 BOX 888 | Hornsund | 76.988 | 15.820 | 07/2019 | surface sediment | 0-1 | 203 | 1.26 | 0.15 | 0.09 | -25.0 | -629.1 | -14.9 | 10.81 | 2.13 |
| HH19 BOX 891 | Hornsund | 76.989 | 16.305 | 07/2019 | surface sediment | 0-1 | 115 | 2.09 | 0.13 | 0.05 | -25.1 | -837.7 | -13.6 | 7.30 | 1.96 |
| 1464 | Hornsund | 77.153 | 16.325 | 07/2015 | surface sediment | 0-5 | 156 | 0.53^a^ | 0.05^a^ | 0.01 | -26.9^a^ | -813.1 | -24.9^b^ | 11.97 | 2.14 |
| 1466 | Hornsund | 77.007 | 16.489 | 07/2015 | surface sediment | 0-5 | 119 | 0.66^a^ | 0.06^a^ | 0.02 | -26.6^a^ | -860.9 | -23.7^b^ | 7.36 | 1.53 |
| 1468 | Hornsund | 77.161 | 16.561 | 07/2015 | surface sediment | 0-5 | 78 | 1.48^a^ | 0.09^a^ | 0.02 | -26.4^a^ | -954.0 | -12.8^b^ | 9.10 | 2.07 |
| 1469 | Hornsund | 77.066 | 16.616 | 07/2015 | surface sediment | 0-5 | 45 | 1.09^a^ | 0.09^a^ | 0.04 | -25.9^a^ | NA | -20.7^b^ | 7.33 | 1.68 |
| 1472 | Hornsund | 77.649 | 16.321 | 07/2015 | surface sediment | 0-5 | 83 | 1.75^a^ | 0.12^a^ | 0.05 | -25.0^a^ | NA | -15.0^b^ | 7.59 | 1.76 |
| 1474 | Hornsund | 77.055 | 16.465 | 07/2015 | surface sediment | 0-5 | 44 | 2.80^a^ | 0.16^a^ | 0.04 | -25.7^a^ | -932.0 | -12.7^b^ | 6.56 | 1.91 |
| 1475 | Hornsund | 77.035 | 16.507 | 07/2015 | surface sediment | 0-5 | 50 | 2.43^a^ | 0.14^a^ | 0.04 | -26.5^a^ | -920.3 | -12.6^b^ | 6.03 | 1.92 |
| 1476 | Hornsund | 77.009 | 16.527 | 07/2015 | surface sediment | 0-5 | 84 | 2.11^a^ | 0.13^a^ | 0.04 | -24.9^a^ | NA | -13.0^b^ | 9.76 | 1.37 |
| 1478 | Hornsund | 77.653 | 16.679 | 07/2015 | surface sediment | 0-5 | 120 | 2.06^a^ | 0.13^a^ | 0.04 | -25.5^a^ | NA | -13.1^b^ | 6.27 | 1.85 |
| 1479 | Hornsund | 77.026 | 16.760 | 07/2015 | surface sediment | 0-5 | 138 | 2.12^a^ | 0.12^a^ | 0.04 | -25.3^a^ | NA | -11.7^b^ | 5.82 | 1.88 |
| 1480 | Hornsund | 77.056 | 16.846 | 07/2015 | surface sediment | 0-5 | 87 | 2.05^a^ | 0.12^a^ | 0.03 | -25.2^a^ | -939.1 | -10.1^b^ | 5.73 | 1.93 |
| 1482 | Hornsund | 77.092 | 17.089 | 07/2015 | surface sediment | 0-5 | 71 | 1.84^a^ | 0.10^a^ | 0.03 | -25.4^a^ | -961.2 | -10.4^b^ | 8.51 | 2.05 |
| 1484 | Hornsund | 77.596 | 16.973 | 07/2015 | surface sediment | 0-5 | 65 | 1.66^a^ | 0.09^a^ | 0.03 | -24.8^a^ | NA | -10.8^b^ | 14.03 | 2.15 |
| 1485 | Hornsund | 77.642 | 16.868 | 07/2015 | surface sediment | 0-5 | 71 | 2.07^a^ | 0.12^a^ | 0.02 | -24.9^a^ | NA | -11.3^b^ | 6.33 | 1.84 |
| 1486 | Hornsund | 77.632 | 16.594 | 07/2015 | surface sediment | 0-5 | 37 | 1.78^a^ | 0.12^a^ | 0.03 | -25.8^a^ | NA | -14.9^b^ | 8.45 | 1.79 |
| 1488 | Hornsund | 77.522 | 16.550 | 07/2015 | surface sediment | 0-5 | 89 | 1.20^a^ | 0.09^a^ | 0.01 | -26.9^a^ | NA | -17.4^b^ | 10.94 | 1.78 |
| 1489 | Hornsund | 77.572 | 16.455 | 07/2015 | surface sediment | 0-5 | 140 | 1.25^a^ | 0.11^a^ | 0.01 | -25.8^a^ | NA | -17.0^b^ | 9.34 | 1.83 |
| 1490 | Hornsund | 77.564 | 16.391 | 07/2016 | surface sediment | 0-5 | 64 | 1.43^a^ | 0.11^a^ | 0.03 | -25.7^a^ | NA | -16.3^b^ | 8.34 | 1.62 |
| 1223 | Hornsund | 76.977 | 15.864 | 07/2016 | surface sediment | 0-1 | 193 | 1.73^a^ | 0.17^a^ | 0.10 | -24.3^a^ | NA | -16.5^b^ | NA | NA |
| 1227 | Hornsund | 77.000 | 15.997 | 07/2016 | surface sediment | 0-1 | 109 | 1.45^a^ | 0.13^a^ | 0.07 | -24.7^a^ | NA | -18.3^b^ | NA | NA |
| 1230 | Hornsund | 76.983 | 16.273 | 07/2016 | surface sediment | 0-1 | 110 | 1.73^a^ | 0.13^a^ | 0.03 | -25.2^a^ | NA | -14.8^b^ | NA | NA |
| Koller #1_1B | Krossfjorden | 79.287 | 12.046 | 07/2015 | surface sediment | 0-2 | 21 | 0.33 | 0.04 | 0.03 | -19.3 | -603.6 | -13.9 | 8.55 | 1.49 |
| Mayerbukta #1_1B | Krossfjorden | 79.266 | 12.160 | 07/2015 | surface sediment | 0-2 | 7 | 0.21 | 0.01 | 0.01 | -18.2 | -699.7 | -14.3 | 16.26 | 1.79 |
| Julibreen #1_1B | Krossfjorden | 79.123 | 11.846 | 07/2015 | surface sediment | 0-2 | 23 | 0.35 | 0.03 | 0.02 | -19.8 | -609.5 | -14.9 | 15.85 | 1.65 |
| 1182 | Dicksonfjorden | 78.805 | 15.362 | 07/2016 | surface sediment | 0-1 | 40 | 0.12^a^ | 0.08 | 0.05 | -25.1^a^ | -741.4 | -13.7^b^ | NA | NA |
| 1193 | Dicksonfjorden | 78.646 | 15.290 | 07/2016 | surface sediment | 0-1 | 109 | 0.81^a^ | 0.11 | 0.07 | -24.0^a^ | -606.1 | -13.4^b^ | NA | NA |

Table S2. Sample information and data of the OC sources considered in this study. Note that “a” and “b” indicate the data published by Kim et al.^[15]^ and Jung et al.^[16]^, respectively. NA and DL denote “not available” and “detection limit”, respectively.

| Sample name | Location | Latitude | Longitude | Sampling date | Source type | TOC | (Ad/Al)v | 3,5-Bd/V | λ | S/V | C/V | δ^13^C_org_ | Δ^14^C_org_ | Reference |
| --- | --- | --- | --- | --- | --- | --- | --- | --- | --- | --- | --- | --- | --- | --- |
|  |  |  |  | (mm/yyyy) |  | (wt. %) |  |  | (mg/g OC) |  |  | (‰ VPDB) | (‰) |  |
| Coal | Ny-Ålesund | 78.923 | 11.917 | 07/2009 | Petrogenic | 42.9 | NA | NA | NA | NA | NA | -25.5 | -1000 | Kim et al.^[4]^ |
| ST3 coal | Ny-Ålesund | 78.928 | 11.906 | 07/2022 | Petrogenic | 44.6 | 0.348 | 0.51 | 0.011 | 0.061 | 0.072 | -23.2 | NA | This study |
| ST4-1 coal | Ny-Ålesund | 78.918 | 11.959 | 07/2022 | Petrogenic | 51.5 | 0.474 | 0.326 | 0.020 | 0.110 | 0.051 | -23.7 | NA | This study |
| ST4-2 coal | Ny-Ålesund | 78.917 | 11.955 | 07/2022 | Petrogenic | 58.6 | 0.659 | 0.585 | 0.013 | 0.047 | 0.050 | -23.2 | NA | This study |
| SK01 (sandstone) | Skansen, Isfjorden-Billefjorden | 78.545 | 16.183 | NA | Petrogenic | 0.1 | DL | DL | DL | DL | DL | -26.4 | NA | This study |
| 080302 (red sandstone) | Halvdanpiggen,Woodfjorden | 79.389 | 13.622 | NA | Petrogenic | DL | DL | DL | DL | DL | DL | DL | NA | This study |
| 13071005 (red sanstone) | Brøggerhalvøya,Kongsfjorden | 78.953 | 11.758 | NA | Petrogenic | DL | DL | DL | DL | DL | DL | DL | NA | This study |
| SYB05 (limestone) | Brøggerhalvøya,Kongsfjorden | 78.944 | 11.724 | NA | Petrogenic | DL | DL | DL | DL | DL | DL | DL | NA | This study |
| SYB56 (dolostone) | Brøggerhalvøya,Kongsfjorden | 78.941 | 11.703 | NA | Petrogenic | DL | DL | DL | DL | DL | DL | DL | NA | This study |
| SK20 (dolostone) | Skansen, Isfjorden-Billefjorden | 78.539 | 16.159 | NA | Petrogenic | DL | DL | DL | DL | DL | DL | DL | NA | This study |
| SYB40.1 (calcareous shale) | Brøggerhalvøya,Kongsfjorden | 78.942 | 11.704 | NA | Petrogenic | 0.2 | DL | DL | DL | DL | DL | -27.0 | NA | This study |
| SF00.5 (chert) | Brøggerhalvøya,Kongsfjorden | 78.915 | 11.654 | NA | Petrogenic | 0.1 | DL | DL | DL | DL | DL | -26.5 | NA | This study |
| 080109 (black mudstone) | Festningen, Isfjorden-Grønfjorden | 78.091 | 13.81 | NA | Petrogenic | 0.3 | DL | DL | DL | DL | DL | -24.4 | NA | This study |
| Sval central (anhydrite) | Skansen, Isfjorden-Billefjorden | 78.545 | 16.183 | NA | Petrogenic | DL | DL | DL | DL | DL | DL | DL | NA | This study |
| KIGAM#25 (shale) | Rotundafjellet, Isfjorden-Billefjorden | 78.475 | 15.812 | NA | Petrogenic | 0.1 | DL | DL | DL | DL | DL | -28.3 | NA | This study |
| 080111 (black mudstone) | Festningen, Isfjorden-Grønfjorden | 78.094 | 13.837 | NA | Petrogenic | 0.5 | DL | DL | DL | DL | DL | -26.4 | NA | This study |
| KIGAM#16 (shale) | Festningen, Isfjorden-Grønfjorden | 78.098 | 13.913 | NA | Petrogenic | 1.1 | DL | DL | DL | DL | DL | -25.3 | NA | This study |
| 080114 (sandstone) | Festningen, Isfjorden-Grønfjorden | 78.099 | 13.948 | NA | Petrogenic | 0.1 | DL | DL | DL | DL | DL | -29.0 | NA | This study |
| A1-1 | Longyearbyen (5-10cm) | 78.171 | 16.033 | 07/2021 | Soil | 15.4 | 3.364 | 0.368 | 11.962 | 0.452 | 0.020 | -29.0 | NA | This study |
| A1-2 | Longyearbyen (5-10cm) | 78.171 | 16.033 | 07/2021 | Soil | 11.5 | 3.450 | 0.340 | 12.407 | 0.433 | 0.026 | -30.2 | NA | This study |
| A1-3 | Longyearbyen (5-10cm) | 78.171 | 16.033 | 07/2021 | Soil | 15.2 | 3.526 | 0.367 | 12.237 | 0.524 | 0.019 | -28.1 | NA | This study |
| A2-1 | Longyearbyen (5-10cm) | 78.172 | 16.022 | 07/2021 | Soil | 12.6 | 4.054 | 0.335 | 8.397 | 0.314 | 0.022 | -28.1 | NA | This study |
| A2-2 | Longyearbyen (5-10cm) | 78.172 | 16.022 | 07/2021 | Soil | 12.4 | 4.235 | 0.344 | 8.460 | 0.323 | 0.022 | -27.1 | NA | This study |
| A2-3 | Longyearbyen (5-10cm) | 78.172 | 16.022 | 07/2021 | Soil | 12.1 | 5.158 | 0.416 | 8.899 | 0.278 | 0.040 | -26.3 | NA | This study |
| B-1 | Longyearbyen (5-10cm) | 78.168 | 16.05 | 07/2021 | Soil | 14.2 | 1.630 | 0.274 | 11.436 | 0.354 | 0.020 | -27.4 | NA | This study |
| B-2 | Longyearbyen (5-10cm) | 78.168 | 16.05 | 07/2021 | Soil | 17.1 | 1.621 | 0.284 | 11.191 | 0.363 | 0.020 | -27.7 | NA | This study |
| B-3 | Longyearbyen (5-10cm) | 78.168 | 16.05 | 07/2021 | Soil | 14.3 | 2.111 | 0.357 | 11.867 | 0.350 | 0.073 | -27.0 | NA | This study |
| D-1 | Longyearbyen (5-10cm) | 78.173 | 16.082 | 07/2021 | Soil | 5.4 | 0.393 | 0.120 | 4.358 | 0.423 | 0.101 | -25.3 | NA | This study |
| D-2 | Longyearbyen (5-10cm) | 78.173 | 16.082 | 07/2021 | Soil | 4.2 | 0.429 | 0.125 | 4.496 | 0.408 | 0.101 | -25.5 | NA | This study |
| D-3 | Longyearbyen (5-10cm) | 78.173 | 16.082 | 07/2021 | Soil | 11.1 | 0.501 | 0.149 | 5.151 | 0.369 | 0.323 | -25.6 | NA | This study |
| NA1 | around Kross/Kongsfjorden (0-10cm) | 78.922 | 11.934 | 07/2009 | Soil | 5.4 | NA | NA | NA | NA | NA | -25.8 | -354.2 | Kim et al.^[4]^ |
| NA2 | around Kross/Kongsfjorden (0-10cm) | 78.921 | 11.918 | 07/2009 | Soil | 2.2 | NA | NA | NA | NA | NA | -26.3 | NA | Kim et al.^[4]^ |
| MP1 | around Kross/Kongsfjorden (0-10cm) | 79.185 | 11.169 | 07/2009 | Soil | 1.5 | NA | NA | NA | NA | NA | -25.4 | NA | Kim et al.^[4]^ |
| MP2 | around Kross/Kongsfjorden (0-10cm) | 79.189 | 11.193 | 07/2009 | Soil | 0.6 | NA | NA | NA | NA | NA | -25.2 | NA | Kim et al.^[4]^ |
| MP3 | around Kross/Kongsfjorden (0-10cm) | 79.201 | 11.267 | 07/2009 | Soil | 1.8 | NA | NA | NA | NA | NA | -26.0 | 107.1 | Kim et al.^[4]^ |
| MP4 | around Kross/Kongsfjorden (0-10cm) | 79.202 | 11.303 | 07/2009 | Soil | 1.4 | NA | NA | NA | NA | NA | -26.1 | NA | Kim et al.^[4]^ |
| MP5 | around Kross/Kongsfjorden (0-10cm) | 79.2 | 11.317 | 07/2009 | Soil | 0.6 | NA | NA | NA | NA | NA | -25.3 | NA | Kim et al.^[4]^ |
| MF1 | around Kross/Kongsfjorden (0-10cm) | 79 | 12 | 07/2009 | Soil | 5.4 | NA | NA | NA | NA | NA | -26.6 | NA | Kim et al.^[4]^ |
| MF2 | around Kross/Kongsfjorden (0-10cm) | 79 | 12 | 07/2009 | Soil | 3.6 | NA | NA | NA | NA | NA | -27.1 | 63.9 | Kim et al.^[4]^ |
| MF3 | around Kross/Kongsfjorden (0-10cm) | 79 | 12 | 07/2009 | Soil | 0.8 | NA | NA | NA | NA | NA | -25.4 | NA | Kim et al.^[4]^ |
| S1 | around Kross/Kongsfjorden (0-10cm) | 78.901 | 12.127 | 07/2009 | Soil | 1.5 | NA | NA | NA | NA | NA | -26.3 | NA | Kim et al.^[4]^ |
| S2 | around Kross/Kongsfjorden (0-10cm) | 78.901 | 12.127 | 07/2009 | Soil | 1.5 | NA | NA | NA | NA | NA | -26.8 | NA | Kim et al.^[4]^ |
| S3 | around Kross/Kongsfjorden (0-10cm) | 78.901 | 12.146 | 07/2009 | Soil | 8.9 | NA | NA | NA | NA | NA | -26.3 | -20.1 | Kim et al.^[4]^ |
| S4 | around Kross/Kongsfjorden (0-10cm) | 78.901 | 12.145 | 07/2009 | Soil | 10.3 | NA | NA | NA | NA | NA | -26.5 | NA | Kim et al.^[4]^ |
| S5 | around Kross/Kongsfjorden (0-10cm) | 78.898 | 12.189 | 07/2009 | Soil | 0.6 | NA | NA | NA | NA | NA | -26.9 | NA | Kim et al.^[4]^ |
| 177 | Midtre Lovenbreen (0-5 cm) | 78.900 | 12.101 | 07/2014 | Soil | 0.25^a^ | 0.45 | 0.244 | 0.137 | 0.241 | 0.058 | -24.3 | NA | This study |
| 149 | Midtre Lovenbreen (0-5 cm) | 78.904 | 12.111 | 07/2014 | Soil | 0.07^a^ | 0.356 | 0.149 | 0.460 | 0.240 | 0.012 | -17.5 | NA | This study |
| 120 | Midtre Lovenbreen (0-5 cm) | 78.902 | 12.073 | 07/2014 | Soil | 0.96^a^ | 0.265 | 0.268 | 3.052 | 0.146 | 0.103 | -25.0 | NA | This study |
| 182 | Midtre Lovenbreen (0-5 cm) | 78.900 | 12.072 | 07/2014 | Soil | 0.29^a^ | 0.303 | 0.340 | 0.869 | 0.173 | 0.166 | -24.5 | NA | This study |
| 191 | Midtre Lovenbreen (0-5 cm) | 78.899 | 12.065 | 07/2014 | Soil | 0.41^a^ | 0.390 | 0.893 | 2.180 | 0.169 | 0.261 | -26.3 | NA | This study |
| 301 | Midtre Lovenbreen (0-5 cm) | 78.897 | 12.063 | 07/2014 | Soil | 0.24^a^ | 0.645 | 1.207 | 0.398 | 0.431 | 0.095 | -23.4 | NA | This study |
| 302 | Midtre Lovenbreen (0-5 cm) | 78.898 | 12.052 | 07/2014 | Soil | 0.14^a^ | 0.643 | 1.147 | 0.193 | 0.708 | 0.095 | -22.7 | NA | This study |
| 303 | Midtre Lovenbreen (0-5 cm) | 78.896 | 12.048 | 07/2014 | Soil | 0.11^a^ | 0.874 | 0.365 | 0.316 | 0.143 | 0.03 | -19.3 | NA | This study |
| Low | Vestre Lovenbreen (5-10 cm) | NA | NA | 07/2011 | Soil | 21.5^b^ | 0.485 | 0.301 | 0.182 | 0.264 | 0.088 | -26.4 | -340.9 | This study |
| Mid | Vestre Lovenbreen (5-10 cm) | NA | NA | 07/2011 | Soil | 85.1^b^ | 0.363 | 0.178 | 0.86 | 0.327 | 0.051 | -26.3 | -251.7 | This study |
| High | Vestre Lovenbreen (5-10 cm) | NA | NA | 07/2011 | Soil | 20.6^b^ | 0.371 | 0.221 | 0.379 | 0.262 | 0.074 | -26.2 | -311.6 | This study |
| 177 | Midtre Lovenbreen (20-30 cm) | 78.9 | 12.101 | 07/2014 | Soil | 0.15 | 0.619 | 0.127 | 0.785 | 0.090 | 0.019 | -23 | NA | This study |
| 120 | Midtre Lovenbreen (20-30 cm) | 78.902 | 12.073 | 07/2014 | Soil | 0.15 | 0.493 | 0.074 | 1.460 | 0.033 | 0.029 | -21.9 | NA | This study |
| 182 | Midtre Lovenbreen (20-30 cm) | 78.900 | 12.072 | 07/2014 | Soil | 0.32 | 0.432 | 0.160 | 0.527 | 0.176 | 0.024 | -23.3 | NA | This study |
| 191 | Midtre Lovenbreen (20-30 cm) | 78.899 | 12.065 | 07/2014 | Soil | 0.18 | 0.590 | 0.095 | 1.132 | 0.060 | 0.043 | -24.4 | NA | This study |
| 301 | Midtre Lovenbreen (20-30 cm) | 78.897 | 12.063 | 07/2014 | Soil | 0.26 | 0.714 | 0.311 | 0.405 | 0.216 | 0.049 | -22.3 | NA | This study |
| Soil | Leirhaugen (0-30 cm) | 78.924 | 11.838 | 2007 | Soil | NA | NA | NA | NA | NA | NA | -25.1 | -517.5 | Kusch et al.^[5]^ |
| Soil | Leirhaugen (30-60 cm) | 78.924 | 11.838 | 2007 | Soil | NA | NA | NA | NA | NA | NA | -25.3 | -858.0 | Kusch et al.^[5]^ |
| Soil | Leirhaugen (60-85 cm) | 78.924 | 11.838 | 2007 | Soil | NA | NA | NA | NA | NA | NA | -25.5 | -960.8 | Kusch et al.^[5]^ |
| Cassiope-1 | Longyearbyen | 78.171 | 16.033 | 07/2021 | Plant | 46.8 | 0.455 | 0.358 | 44.387 | 0.061 | 0.429 | -31.0 | NA | This study |
| Cassiope-2 | Longyearbyen | 78.171 | 16.033 | 07/2021 | Plant | 48.8 | 0.459 | 0.286 | 62.181 | 0.363 | 3.322 | -32.6 | NA | This study |
| Cassiope-3 | Longyearbyen | 78.171 | 16.033 | 07/2021 | Plant | 51.3 | 0.459 | 0.201 | 62.189 | 0.013 | 0.359 | -31.0 | NA | This study |
| Salix polaris-1 | Longyearbyen | 78.172 | 16.022 | 07/2021 | Plant | 44.7 | 0.285 | 0.105 | 38.091 | 0.406 | 0.770 | -31.4 | NA | This study |
| Salix polaris-2 | Longyearbyen | 78.172 | 16.022 | 07/2021 | Plant | 46.4 | 0.285 | 0.124 | 19.798 | 0.259 | 0.550 | -32.9 | NA | This study |
| Salix polaris-3 | Longyearbyen | 78.172 | 16.022 | 07/2021 | Plant | 54.3 | 0.283 | 0.136 | 19.001 | 0.170 | 1.731 | -30.9 | NA | This study |
| Moss-1 | Longyearbyen | 78.168 | 16.05 | 07/2021 | Plant | 41.6 | 0.315 | 0.324 | 6.189 | 0.091 | 0.381 | -29.8 | NA | This study |
| Moss-2 | Longyearbyen | 78.168 | 16.05 | 07/2021 | Plant | 41.5 | 0.370 | 0.402 | 8.134 | 0.339 | 1.397 | -30.3 | NA | This study |
| Moss-3 | Longyearbyen | 78.168 | 16.05 | 07/2021 | Plant | 40.8 | 0.370 | 0.243 | 8.150 | 0.031 | 0.214 | -28.8 | NA | This study |
| Dryas-1 | Longyearbyen | 78.173 | 16.082 | 07/2021 | Plant | 48.3 | 0.247 | 0.490 | 11.392 | 0.078 | 0.477 | -31.6 | NA | This study |
| Dryas-2 | Longyearbyen | 78.173 | 16.082 | 07/2021 | Plant | 48.0 | 0.229 | 0.369 | 16.63 | 0.438 | 2.545 | -35.2 | NA | This study |
| Dryas-3 | Longyearbyen | 78.173 | 16.082 | 07/2021 | Plant | 60.4 | 0.229 | 0.338 | 16.74 | 0.026 | 0.187 | -31.1 | NA | This study |
| ALT (n=48) | Alert (Canadian high Arctic) | 82.499 | -62.342 | 2015-2019 | Atmospheric CO_2_ | NA | NA | NA | NA | NA | NA | NA | 9.1±6.4 | Levin et al.^[8]^ |
| POC (n=15) | Isfjorden/Hornsund/Kongsfjorden | 75-80 | 10-15 | 06/2012 | Marine | NA | NA | NA | NA | NA | NA | -22.5±1.8 | NA | Holding et al.^[9]^ |
| Marine phytobenthos and plankton (n=5) | Kongsfjorden | 78.59 | 11.58 | 08/2011 | Marine | NA | NA | NA | NA | NA | NA | -19.0  (-23.9 ~ -15.8) | NA | Kuliński et al.^[10]^ |
| POC (n=21) | the northwestern part of the Svalbard Archipelago | NA | NA | 05/2014 | Marine | NA | NA | NA | NA | NA | NA | NA | 5.18±6.27 | Sanz-Martin et al.^[13]^ |

Table S3. Lignin phenol data obtained from fjord surface sediments in this study. Note that no samples from the Woodfjorden were available for the lignin phenols analyses. NA and DL denote “not available” and “detection limit”, respectively.

| Sample name | Location | Latitude | Longitude | Sample type | Vl (mg/g OC) | Vn (mg/g OC) | Sl (mg/g OC) | Sn (mg/g OC) | Vd (mg/g OC) | Sd (mg/g OC) | pCd (mg/g OC) | Fd (mg/g OC) | λ  (mg/g OC) | 3,5-Bd (mg/g OC) |
| --- | --- | --- | --- | --- | --- | --- | --- | --- | --- | --- | --- | --- | --- | --- |
| HH17-1107 | Wijdefjorden | 79.000 | 16.220 | surface sediment | 0.099 | 0.039 | 0.032 | 0.008 | 0.078 | 0.017 | 0.049 | 0.024 | 0.346 | 0.232 |
| HH17-1108 | Wijdefjorden | 79.160 | 15.960 | surface sediment | 0.085 | 0.039 | 0.025 | 0.013 | 0.084 | 0.007 | 0.041 | 0.017 | 0.310 | 0.469 |
| HH17-1109 | Wijdefjorden | 79.300 | 15.780 | surface sediment | 0.087 | 0.043 | 0.046 | 0.016 | 0.114 | 0.026 | 0.056 | 0.033 | 0.423 | 0.281 |
| HH17-1110 | Wijdefjorden | 79.740 | 15.420 | surface sediment | 0.111 | 0.048 | 0.047 | 0.011 | 0.223 | 0.026 | 0.024 | 0.034 | 0.524 | 0.755 |
| HH17-1111 | Wijdefjorden | 79.870 | 15.380 | surface sediment | 0.112 | 0.042 | 0.047 | 0.024 | 0.173 | 0.023 | 0.017 | 0.015 | 0.453 | 0.459 |
| HH17-1086 | Wijdefjorden | 80.270 | 16.210 | surface sediment | 0.080 | 0.041 | 0.024 | 0.020 | 0.117 | 0.015 | 0.025 | 0.013 | 0.335 | 0.318 |
| HH12-964GC | Woodfjorden | 79.650 | 13.750 | surface sediment | NA | NA | NA | NA | NA | NA | NA | NA | NA | NA |
| HH12-969GC | Woodfjorden | 79.350 | 13.960 | surface sediment | NA | NA | NA | NA | NA | NA | NA | NA | NA | NA |
| HH19 BOX 847 | Isfjorden | 78.334 | 15.331 | surface sediment | 0.031 | 0.011 | 0.008 | 0.004 | 0.020 | 0.004 | 0.006 | 0.011 | 0.094 | 0.087 |
| HH19 BOX 850 | Isfjorden | 78.153 | 13.741 | surface sediment | 0.032 | 0.011 | 0.007 | 0.003 | 0.023 | 0.003 | 0.014 | 0.014 | 0.108 | 0.144 |
| HH19 BOX 855 | van Mijenfjorden | 77.856 | 16.658 | surface sediment | 0.010 | 0.003 | 0.001 | 0.001 | 0.005 | 0.001 | 0.002 | 0.002 | 0.025 | 0.023 |
| HH19 BOX 858 | van Mijenfjorden | 77.826 | 16.548 | surface sediment | DL | DL | DL | DL | DL | DL | DL | DL | DL | DL |
| HH19 BOX 863 | van Mijenfjorden | 77.786 | 16.006 | surface sediment | 0.022 | 0.017 | 0.005 | 0.003 | 0.013 | 0.003 | 0.012 | 0.007 | 0.084 | 0.078 |
| HH19 BOX 868 | van Mijenfjorden | 77.734 | 14.873 | surface sediment | 0.023 | 0.010 | 0.007 | 0.003 | 0.014 | 0.003 | 0.011 | 0.009 | 0.079 | 0.056 |
| HH19 BOX 872 | van Mijenfjorden | 77.567 | 15.287 | surface sediment | DL | DL | DL | DL | DL | DL | DL | DL | DL | DL |
| HH19 BOX 876 | van Mijenfjorden | 77.609 | 14.579 | surface sediment | DL | DL | DL | DL | DL | DL | DL | DL | DL | DL |
| HH19 BOX 895 | Hambergbukta | 77.045 | 17.163 | surface sediment | 0.014 | 0.004 | 0.002 | 0.001 | 0.006 | 0.001 | 0.009 | 0.003 | 0.040 | 0.040 |
| HH19 BOX 898 | Hambergbukta | 76.997 | 17.354 | surface sediment | 0.041 | 0.004 | 0.004 | 0.007 | 0.018 | 0.003 | 0.032 | 0.090 | 0.200 | 0.236 |
| HH19 BOX 900 | Hambergbukta | 76.982 | 17.829 | surface sediment | 0.015 | 0.003 | 0.003 | 0.002 | 0.010 | 0.001 | 0.009 | 0.013 | 0.057 | 0.082 |
| HH19 BOX 885 | Hornsund | 76.937 | 15.390 | surface sediment | 0.020 | 0.009 | 0.008 | 0.003 | 0.007 | 0.001 | 0.005 | 0.002 | 0.055 | 0.145 |
| HH19 BOX 888 | Hornsund | 76.988 | 15.820 | surface sediment | 0.018 | 0.004 | 0.007 | 0.007 | 0.020 | 0.002 | 0.008 | 0.001 | 0.067 | 0.101 |
| HH19 BOX 891 | Hornsund | 76.989 | 16.305 | surface sediment | DL | DL | DL | DL | DL | DL | DL | DL | DL | DL |
| 1464 | Hornsund | 77.153 | 16.325 | surface sediment | 0.010 | 0.005 | 0.007 | 0.006 | 0.015 | 0.004 | 0.005 | 0.003 | 0.055 | 0.042 |
| 1466 | Hornsund | 77.007 | 16.489 | surface sediment | 0.018 | 0.003 | 0.006 | 0.000 | 0.008 | 0.000 | 0.000 | 0.000 | 0.035 | 0.012 |
| 1468 | Hornsund | 77.161 | 16.561 | surface sediment | 0.015 | 0.001 | 0.001 | 0.000 | 0.003 | 0.000 | 0.001 | 0.000 | 0.021 | 0.008 |
| 1469 | Hornsund | 77.066 | 16.616 | surface sediment | DL | DL | DL | DL | DL | DL | DL | DL | DL | DL |
| 1472 | Hornsund | 77.649 | 16.321 | surface sediment | DL | DL | DL | DL | DL | DL | DL | DL | DL | DL |
| 1474 | Hornsund | 77.055 | 16.465 | surface sediment | 0.002 | 0.002 | 0.001 | 0.001 | 0.002 | 0.000 | 0.001 | 0.000 | 0.010 | 0.017 |
| 1475 | Hornsund | 77.035 | 16.507 | surface sediment | 0.003 | 0.003 | 0.001 | 0.001 | 0.003 | 0.000 | 0.002 | 0.001 | 0.014 | 0.022 |
| 1476 | Hornsund | 77.009 | 16.527 | surface sediment | DL | DL | DL | DL | DL | DL | DL | DL | DL | DL |
| 1478 | Hornsund | 77.653 | 16.679 | surface sediment | 0.001 | 0.000 | 0.000 | 0.000 | 0.000 | 0.000 | 0.000 | 0.000 | 0.001 | 0.003 |
| 1479 | Hornsund | 77.026 | 16.760 | surface sediment | 0.006 | 0.001 | 0.000 | 0.000 | 0.002 | 0.000 | 0.000 | 0.022 | 0.032 | 0.007 |
| 1480 | Hornsund | 77.056 | 16.846 | surface sediment | DL | DL | DL | DL | DL | DL | DL | DL | DL | DL |
| 1482 | Hornsund | 77.092 | 17.089 | surface sediment | 0.013 | 0.001 | 0.003 | 0.000 | 0.004 | 0.000 | 0.002 | 0.000 | 0.024 | 0.015 |
| 1484 | Hornsund | 77.596 | 16.973 | surface sediment | 0.011 | 0.001 | 0.001 | 0.000 | 0.001 | 0.000 | 0.000 | 0.000 | 0.014 | 0.013 |
| 1485 | Hornsund | 77.642 | 16.868 | surface sediment | DL | DL | DL | DL | DL | DL | DL | DL | DL | DL |
| 1486 | Hornsund | 77.632 | 16.594 | surface sediment | DL | DL | DL | DL | DL | DL | DL | DL | DL | DL |
| 1488 | Hornsund | 77.522 | 16.550 | surface sediment | 0.013 | 0.002 | 0.001 | 0.001 | 0.003 | 0.000 | 0.024 | 0.000 | 0.045 | 0.076 |
| 1489 | Hornsund | 77.572 | 16.455 | surface sediment | DL | DL | DL | DL | DL | DL | DL | DL | DL | DL |
| 1490 | Hornsund | 77.564 | 16.391 | surface sediment | DL | DL | DL | DL | DL | DL | DL | DL | DL | DL |
| 1223 | Hornsund | 76.977 | 15.864 | surface sediment | DL | DL | DL | DL | DL | DL | DL | DL | DL | DL |
| 1227 | Hornsund | 77.000 | 15.997 | surface sediment | DL | DL | DL | DL | DL | DL | DL | DL | DL | DL |
| 1230 | Hornsund | 76.983 | 16.273 | surface sediment | 0.014 | 0.001 | 0.003 | 0.000 | 0.013 | 0.001 | 0.004 | 0.000 | 0.037 | 0.035 |
| Koller #1_1B | Krossfjorden | 79.287 | 12.046 | surface sediment | 0.074 | 0.028 | 0.034 | 0.014 | 0.074 | 0.011 | 0.036 | 0.023 | 0.294 | 0.594 |
| Mayerbukta #1_1B | Krossfjorden | 79.266 | 12.160 | surface sediment | 0.040 | 0.014 | 0.026 | 0.008 | 0.039 | 0.009 | 0.015 | 0.019 | 0.169 | 0.171 |
| Julibreen #1_1B | Krossfjorden | 79.123 | 11.846 | surface sediment | 0.056 | 0.024 | 0.049 | 0.024 | 0.065 | 0.017 | 0.031 | 0.031 | 0.298 | 0.089 |
| 1182 | Dicksonfjorden | 78.805 | 15.362 | surface sediment | DL | DL | DL | DL | DL | DL | DL | DL | DL | DL |
| 1193 | Dicksonfjorden | 78.646 | 15.290 | surface sediment | 0.031 | 0.012 | 0.014 | 0.005 | 0.030 | 0.004 | 0.013 | 0.006 | 0.115 | 0.097 |

Table S4. Data compiled from the literature in this study. NA denotes “not available”.

| Sample name | Location | Latitude | Longitude | Sampling date  (mm/yyyy) | Sample type | Sample depth  (cm) | Water depth  (m) | TOC  (wt. %) | N_tot_  (wt. %) | N_org_  (wt. %) | δ^13^C_org_  (‰ VPDB) | Δ^14^C_org_ (‰) | Reference |
| --- | --- | --- | --- | --- | --- | --- | --- | --- | --- | --- | --- | --- | --- |
| 1185 | Dicksonfjorden | 78.757 | 15.301 | 07/2016 | surface sediment | 0-1 | 67 | 0.40 | 0.06 | NA | -24.3 | NA | Kim et al.^[4]^ |
| 1187 | Dicksonfjorden | 78.699 | 15.325 | 07/2016 | surface sediment | 0-1 | 77 | 0.69 | 0.08 | NA | -24.1 | NA | Kim et al.^[4]^ |
| 1190 | Dicksonfjorden | 78.665 | 15.358 | 07/2016 | surface sediment | 0-1 | 97 | 1.06 | 0.12 | NA | -24.2 | NA | Kim et al.^[4]^ |
| 1196 | Dicksonfjorden | 78.787 | 15.299 | 07/2016 | surface sediment | 0-1 | 53 | 0.17 | 0.04 | NA | -25.3 | NA | Kim et al.^[4]^ |
| 1199 | Dicksonfjorden | 78.724 | 15.316 | 07/2016 | surface sediment | 0-1 | 37 | 0.80 | 0.09 | NA | -24.0 | NA | Kim et al.^[4]^ |
| 1202 | Dicksonfjorden | 78.726 | 15.316 | 07/2016 | surface sediment | 0-1 | 87 | 0.63 | 0.06 | NA | -25.0 | NA | Kim et al.^[4]^ |
| I-1 | Kongsfjorden | 79.035 | 11.284 | 08/2014 | surface sediment | 0-1 | 250 | 1.73 | 0.20 | 0.14 | -24.0 | NA | Kumar et al.^[17]^ |
| I-2A | Kongsfjorden | 78.984 | 11.509 | 08/2014 | surface sediment | 0-1 | 320 | 2.10 | 0.20 | NA | -24.4 | NA | Kumar et al.^[17]^ |
| I-2 | Kongsfjorden | 78.993 | 11.555 | 08/2014 | surface sediment | 0-1 | 254 | 7.97 | 0.21 | 0.16 | -23.7 | NA | Kumar et al.^[17]^ |
| I-2B | Kongsfjorden | 79.017 | 11.625 | 08/2014 | surface sediment | 0-1 | 230 | 1.57 | 0.13 | NA | -23.3 | NA | Kumar et al.^[17]^ |
| I-2C | Kongsfjorden | 79.041 | 11.702 | 08/2014 | surface sediment | 0-1 | 70 | 1.01 | 0.08 | NA | -22.9 | NA | Kumar et al.^[17]^ |
| I-3 | Kongsfjorden | 78.976 | 11.692 | 08/2014 | surface sediment | 0-1 | 294 | 1.77 | 0.16 | 0.11 | -22.6 | NA | Kumar et al.^[17]^ |
| I-4 | Kongsfjorden | 78.959 | 11.822 | 08/2014 | surface sediment | 0-1 | 180 | 1.70 | 0.11 | 0.08 | -22.7 | NA | Kumar et al.^[17]^ |
| I-5 | Kongsfjorden | 78.941 | 11.958 | 08/2014 | surface sediment | 0-1 | 302 | 1.36 | 0.13 | 0.08 | -22.9 | NA | Kumar et al.^[17]^ |
| I-6 | Kongsfjorden | 78.923 | 12.094 | 08/2014 | surface sediment | 0-1 | 145 | 0.83 | 0.08 | 0.04 | -23.1 | NA | Kumar et al.^[17]^ |
| I-7 | Kongsfjorden | 78.993 | 12.300 | 08/2014 | surface sediment | 0-1 | 74 | 0.65 | 0.05 | 0.01 | -22.7 | NA | Kumar et al.^[17]^ |
| I-8 | Kongsfjorden | 78.895 | 12.320 | 08/2014 | surface sediment | 0-1 | 45 | 0.52 | 0.03 | 0.01 | -22.5 | NA | Kumar et al.^[17]^ |
| HH14-897-GC-MF | Hornsund | 76.993 | 16.405 | 10/2014 | surface sediment | 0-2 | 125 | 1.9 | NA | NA | -24.6 | -885.2 | Ruben et al.^[12]^ |
| HE560_26-2 | Hornsund | 76.992 | 16.565 | 08/2020 | surface sediment | 0-1 | 46 | 1.5 | NA | NA | -24.8 | -951.5 | Ruben et al.^[12]^ |
| HE519_2-3 | Hornsund | 76.983 | 15.820 | 09/2018 | surface sediment | 0-1 | 202 | 1.2 | NA | NA | -23.8 | -574.4 | Ruben et al.^[12]^ |
| WP280 | Kongsfjorden | 78.933 | 11.870 | 2008 | surface sediment | 0-2 | 3.6 | 0.2 | NA | NA | NA | -914.6 | Kusch et al.^[5]^ |
| 4 | Hornsund | NA | NA | NA | surface sediment | 0-1 | NA | 1.97 | 0.15 | NA | -24.9 | NA | Koziorowska et al.^[18]^ |
| 4 | Hornsund | NA | NA | NA | surface sediment | 1-2 | NA | 1.93 | 0.14 | NA | -24.9 | NA | Koziorowska et al.^[18]^ |
| 5 | Hornsund | NA | NA | NA | surface sediment | 0-1 | NA | 1.73 | 0.19 | NA | -24.1 | NA | Koziorowska et al.^[18]^ |
| 5 | Hornsund | NA | NA | NA | surface sediment | 1-2 | NA | 1.42 | 0.15 | NA | -24.6 | NA | Koziorowska et al.^[18]^ |
| 6 | Hornsund | NA | NA | NA | surface sediment | 0-1 | NA | 1.49 | 0.18 | NA | -23.9 | NA | Koziorowska et al.^[18]^ |
| 6 | Hornsund | NA | NA | NA | surface sediment | 1-2 | NA | 1.43 | 0.16 | NA | -24.3 | NA | Koziorowska et al.^[18]^ |
| 7 | Hornsund | NA | NA | NA | surface sediment | 0-1 | NA | 1.62 | 0.19 | NA | -24.1 | NA | Koziorowska et al.^[18]^ |
| 7 | Hornsund | NA | NA | NA | surface sediment | 1-2 | NA | 1.45 | 0.16 | NA | -24.2 | NA | Koziorowska et al.^[18]^ |
| 11 | Adventfjord | NA | NA | NA | surface sediment | 0-1 | NA | 2.33 | 0.19 | NA | -24.7 | NA | Koziorowska et al.^[18]^ |
| 11 | Adventfjord | NA | NA | NA | surface sediment | 1-2 | NA | 2.35 | 0.17 | NA | -24.9 | NA | Koziorowska et al.^[18]^ |
| 12 | Adventfjord | NA | NA | NA | surface sediment | 0-1 | NA | 2.31 | 0.18 | NA | -24.7 | NA | Koziorowska et al.^[18]^ |
| 12 | Adventfjord | NA | NA | NA | surface sediment | 1-2 | NA | 2.35 | 0.16 | NA | -24.9 | NA | Koziorowska et al.^[18]^ |
| 14 | Adventfjord | NA | NA | NA | surface sediment | 0-2 | NA | 3.85 | 0.14 | NA | -25.9 | NA | Koziorowska et al.^[18]^ |
| Krossfjord IB | Kongsfjorden | 79.295 | 11.622 | 08/2008 | IRD | NA | NA | 0.10 | 0.05 | NA | -20.3 | -862.4 | Kim et al.^[4]^ |
| Kongfjord IB-1 | Kongsfjorden | 78.907 | 12.405 | 08/2008 | IRD | NA | NA | 0.10 | 0.03 | NA | -23.2 | -755.7 | Kim et al.^[4]^ |
| NP-07-13-09 | Kongsfjorden | 79.059 | 10.666 | 08/2008 | surface sediment | 0-1 | 326 | 1.60 | 0.23 | NA | -22.3 | -343.7 | Kim et al.^[4]^ |
| NP-07-13-13 | Kongsfjorden | 79.295 | 11.622 | 08/2008 | surface sediment | 0-1 | 205 | 0.50 | 0.07 | NA | -21.5 | -203 | Kim et al.^[4]^ |
| NP-07-13-16 | Kongsfjorden | 79.186 | 11.761 | 08/2008 | surface sediment | 0-1 | 374 | 1.00 | 0.14 | NA | -21.4 | -273.1 | Kim et al.^[4]^ |
| NP-07-13-21 | Kongsfjorden | 79.225 | 11.924 | 08/2008 | surface sediment | 0-1 | 279 | 1.00 | 0.14 | NA | -21.9 | -210.9 | Kim et al.^[4]^ |
| NP-07-13-51 | Kongsfjorden | 79.044 | 11.370 | 08/2008 | surface sediment | 0-1 | 335 | 1.60 | 0.20 | NA | -22.6 | -361.4 | Kim et al.^[4]^ |
| NP-07-13-60 | Kongsfjorden | 78.928 | 12.062 | 08/2008 | surface sediment | 0-1 | 204 | 0.60 | 0.08 | NA | -23.6 | -415.4 | Kim et al.^[4]^ |
| NP-07-13-61 | Kongsfjorden | 78.907 | 12.405 | 08/2008 | surface sediment | 0-1 | 73 | 0.20 | 0.03 | NA | -21.3 | -735.2 | Kim et al.^[4]^ |
| 1240 | Norwegian Sea | 75.042 | 19.188 | summer 2001 | surface sediment | 0-1 | 94 | 0.53 | 0.07 | 0.05 | NA | NA | Knies et al.^[19]^ |
| 1241 | Norwegian Sea | 74.839 | 17.582 | summer 2001 | surface sediment | 0-1 | 297 | 2.14 | 0.29 | 0.25 | NA | NA | Knies et al.^[19]^ |
| 1242 | Norwegian Sea | 75.550 | 13.344 | summer 2001 | surface sediment | 0-1 | 1297 | 1.07 | 0.16 | 0.14 | NA | NA | Knies et al.^[19]^ |
| 1243 | Norwegian Sea | 76.247 | 16.594 | summer 2001 | surface sediment | 0-1 | 333 | 1.82 | 0.23 | 0.18 | NA | NA | Knies et al.^[19]^ |
| 1244 | Storfjorden | 78.197 | 19.177 | summer 2001 | surface sediment | 0-1 | 96 | 2.17 | 0.24 | 0.14 | NA | NA | Knies et al.^[19]^ |
| 1245 | Storfjorden | 77.753 | 19.139 | summer 2001 | surface sediment | 0-1 | 180 | 2.37 | 0.27 | 0.17 | NA | NA | Knies et al.^[19]^ |
| 1246 | Storfjorden | 76.794 | 19.446 | summer 2001 | surface sediment | 0-1 | 153 | 2.07 | 0.23 | 0.14 | NA | NA | Knies et al.^[19]^ |
| 1249 | Inner shelf | 76.967 | 15.252 | summer 2001 | surface sediment | 0-1 | 156 | 1.91 | 0.23 | 0.14 | NA | NA | Knies et al.^[19]^ |
| 1250 | Hornsund | 76.994 | 15.763 | summer 2001 | surface sediment | 0-1 | 228 | 1.65 | 0.18 | 0.10 | NA | NA | Knies et al.^[19]^ |
| 1251 | van Mijenfjorden | 77.758 | 14.919 | summer 2001 | surface sediment | 0-1 | 115 | 1.84 | 0.17 | 0.08 | NA | NA | Knies et al.^[19]^ |
| 1254 | van Mijenfjorden | 77.858 | 16.591 | summer 2001 | surface sediment | 0-1 | 76 | 2.03 | 0.14 | 0.04 | NA | NA | Knies et al.^[19]^ |
| 1255 | van Mijenfjorden | 77.769 | 15.190 | summer 2001 | surface sediment | 0-1 | 83 | 1.74 | 0.15 | 0.07 | NA | NA | Knies et al.^[19]^ |
| 1258 | van Mijenfjorden | 78.017 | 15.700 | summer 2001 | surface sediment | 0-1 | 43 | 1.72 | 0.14 | 0.05 | NA | NA | Knies et al.^[19]^ |
| 1260 | van Mijenfjorden | 77.636 | 14.216 | summer 2001 | surface sediment | 0-1 | 162 | 1.77 | 0.23 | 0.16 | NA | NA | Knies et al.^[19]^ |
| 1261 | Marine | 77.489 | 10.607 | summer 2001 | surface sediment | 0-1 | 1291 | 0.89 | 0.12 | 0.08 | NA | NA | Knies et al.^[19]^ |
| 1262 | Marine | 77.506 | 11.275 | summer 2001 | surface sediment | 0-1 | 603 | 0.82 | 0.09 | 0.05 | NA | NA | Knies et al.^[19]^ |
| 1263 | Inner shelf | 77.450 | 12.935 | summer 2001 | surface sediment | 0-1 | 196 | 1.96 | 0.24 | 0.16 | NA | NA | Knies et al.^[19]^ |
| 1264 | Inner shelf | 77.564 | 12.608 | summer 2001 | surface sediment | 0-1 | 103 | 1.53 | 0.19 | 0.13 | NA | NA | Knies et al.^[19]^ |
| 1265 | Isfjorden | 78.367 | 16.376 | summer 2001 | surface sediment | 0-1 | 87 | 1.88 | 0.17 | 0.09 | NA | NA | Knies et al.^[19]^ |
| 1266 | Isfjorden | 78.597 | 15.264 | summer 2001 | surface sediment | 0-1 | 256 | 2.24 | 0.28 | 0.20 | NA | NA | Knies et al.^[19]^ |
| 1267 | Isfjorden | 78.150 | 13.834 | summer 2001 | surface sediment | 0-1 | 416 | 2.68 | 0.37 | 0.29 | NA | NA | Knies et al.^[19]^ |
| 1268 | Inner shelf | 78.747 | 11.641 | summer 2001 | surface sediment | 0-1 | 102 | 1.19 | 0.19 | 0.12 | NA | NA | Knies et al.^[19]^ |
| 1269 | Inner shelf | 78.372 | 12.308 | summer 2001 | surface sediment | 0-1 | 169 | 2.00 | 0.25 | 0.16 | NA | NA | Knies et al.^[19]^ |
| 1270 | Inner shelf | 78.339 | 12.311 | summer 2001 | surface sediment | 0-1 | 259 | 2.41 | 0.31 | 0.22 | NA | NA | Knies et al.^[19]^ |
| 1272 | Marine | 78.292 | 8.824 | summer 2001 | surface sediment | 0-1 | 1400 | 1.20 | 0.16 | 0.11 | NA | NA | Knies et al.^[19]^ |
| 1273 | Marine | 78.286 | 9.328 | summer 2001 | surface sediment | 0-1 | 600 | 0.85 | 0.08 | 0.04 | NA | NA | Knies et al.^[19]^ |
| 1274 | Marine | 78.250 | 9.400 | summer 2001 | surface sediment | 0-1 | 430 | 0.89 | 0.10 | 0.05 | NA | NA | Knies et al.^[19]^ |
| 1275 | Marine | 78.503 | 10.216 | summer 2001 | surface sediment | 0-1 | 297 | 2.21 | 0.26 | 0.19 | NA | NA | Knies et al.^[19]^ |
| 1276 | Marine | 78.572 | 10.339 | summer 2001 | surface sediment | 0-1 | 131 | 2.14 | 0.31 | 0.25 | NA | NA | Knies et al.^[19]^ |
| 1277 | Marine | 78.819 | 9.443 | summer 2001 | surface sediment | 0-1 | 404 | 1.44 | 0.18 | 0.13 | NA | NA | Knies et al.^[19]^ |
| 1278 | Marine | 78.586 | 9.038 | summer 2001 | surface sediment | 0-1 | 601 | 1.13 | 0.13 | 0.09 | NA | NA | Knies et al.^[19]^ |
| 1279 | Marine | 78.761 | 7.828 | summer 2001 | surface sediment | 0-1 | 1203 | 1.36 | 0.18 | 0.13 | NA | NA | Knies et al.^[19]^ |
| 1280 | Marine | 78.725 | 8.704 | summer 2001 | surface sediment | 0-1 | 787 | 1.31 | 0.16 | 0.11 | NA | NA | Knies et al.^[19]^ |
| 1281 | Marine | 78.750 | 9.839 | summer 2001 | surface sediment | 0-1 | 106 | 1.29 | 0.17 | 0.13 | NA | NA | Knies et al.^[19]^ |
| 1282 | Marine | 79.175 | 6.817 | summer 2001 | surface sediment | 0-1 | 1400 | 1.18 | 0.16 | 0.12 | NA | NA | Knies et al.^[19]^ |
| 1284 | Marine | 79.200 | 8.438 | summer 2001 | surface sediment | 0-1 | 604 | 0.93 | 0.11 | 0.05 | NA | NA | Knies et al.^[19]^ |
| 1285 | Marine | 78.942 | 9.626 | summer 2001 | surface sediment | 0-1 | 91 | 1.96 | 0.22 | 0.16 | NA | NA | Knies et al.^[19]^ |
| 1286 | Inner shelf | 78.878 | 11.344 | summer 2001 | surface sediment | 0-1 | 159 | 1.13 | 0.17 | 0.12 | NA | NA | Knies et al.^[19]^ |
| 1287 | Kongsfjorden | 79.192 | 11.776 | summer 2001 | surface sediment | 0-1 | 364 | 0.97 | 0.13 | 0.11 | NA | NA | Knies et al.^[19]^ |
| 1288 | Kongsfjorden | 79.197 | 11.827 | summer 2001 | surface sediment | 0-1 | 308 | 1.38 | 0.19 | 0.15 | NA | NA | Knies et al.^[19]^ |
| 1289 | Inner shelf | 79.289 | 10.836 | summer 2001 | surface sediment | 0-1 | 319 | 2.23 | 0.29 | 0.22 | NA | NA | Knies et al.^[19]^ |
| 1290 | Marine | 78.950 | 9.624 | summer 2001 | surface sediment | 0-1 | 250 | 0.95 | 0.13 | 0.10 | NA | NA | Knies et al.^[19]^ |
| 1292 | Marine | 79.183 | 9.337 | summer 2001 | surface sediment | 0-1 | 80 | 1.51 | 0.21 | 0.17 | NA | NA | Knies et al.^[19]^ |
| 1 | Marine | 72.000 | 22.000 | 2003/2004 | surface sediment | 0-1 | 367 | 0.82 | 0.12 | 0.10 | -21.7 | NA | Knies et al.^[11]^ |
| 2 | Marine | 72.020 | 20.920 | 2003/2004 | surface sediment | 0-1 | 371 | 0.92 | 0.14 | 0.11 | -22.0 | NA | Knies et al.^[11]^ |
| 3 | Marine | 72.030 | 19.850 | 2003/2004 | surface sediment | 0-1 | 324 | 1.01 | 0.15 | 0.13 | -21.8 | NA | Knies et al.^[11]^ |
| 4 | Marine | 72.020 | 18.770 | 2003/2004 | surface sediment | 0-1 | 315 | 0.97 | 0.15 | 0.13 | -21.7 | NA | Knies et al.^[11]^ |
| 6 | Marine | 72.020 | 16.620 | 2003/2004 | surface sediment | 0-1 | 362 | 0.37 | 0.06 | 0.05 | -21.9 | NA | Knies et al.^[11]^ |
| 7 | Marine | 72.020 | 15.520 | 2003/2004 | surface sediment | 0-1 | 767 | 0.29 | 0.03 | 0.02 | -23.6 | NA | Knies et al.^[11]^ |
| 9 | Marine | 72.010 | 14.620 | 2003/2004 | surface sediment | 0-1 | 1317 | 0.67 | 0.1 | 0.09 | -21.5 | NA | Knies et al.^[11]^ |
| 11 | Marine | 73.170 | 12.940 | 2003/2004 | surface sediment | 0-1 | 1499 | 0.74 | 0.11 | 0.10 | -21.7 | NA | Knies et al.^[11]^ |
| 12 | Marine | 73.170 | 14.090 | 2003/2004 | surface sediment | 0-1 | 1030 | 0.42 | 0.06 | 0.05 | -22.0 | NA | Knies et al.^[11]^ |
| 13 | Marine | 73.170 | 15.230 | 2003/2004 | surface sediment | 0-1 | 485 | 0.33 | 0.05 | 0.04 | -22.6 | NA | Knies et al.^[11]^ |
| 14 | Marine | 73.170 | 16.380 | 2003/2004 | surface sediment | 0-1 | 475 | 0.79 | 0.11 | 0.08 | -22.3 | NA | Knies et al.^[11]^ |
| 15 | Marine | 73.170 | 17.540 | 2003/2004 | surface sediment | 0-1 | 460 | 0.88 | 0.12 | 0.09 | -22.4 | NA | Knies et al.^[11]^ |
| 16 | Marine | 73.170 | 18.820 | 2003/2004 | surface sediment | 0-1 | 423 | 0.87 | 0.12 | 0.09 | -22.5 | NA | Knies et al.^[11]^ |
| 17 | Marine | 73.170 | 19.860 | 2003/2004 | surface sediment | 0-1 | 441 | 1.15 | 0.14 | 0.12 | -22.9 | NA | Knies et al.^[11]^ |
| 18 | Marine | 73.170 | 20.950 | 2003/2004 | surface sediment | 0-1 | 463 | 1.41 | 0.18 | 0.15 | -22.6 | NA | Knies et al.^[11]^ |
| 19 | Marine | 73.170 | 22.010 | 2003/2004 | surface sediment | 0-1 | 444 | 1.40 | 0.19 | 0.15 | -22.4 | NA | Knies et al.^[11]^ |
| 20 | Marine | 74.820 | 18.020 | 2003/2004 | surface sediment | 0-1 | 296 | 1.97 | 0.25 | 0.21 | -22.4 | NA | Knies et al.^[11]^ |
| 21 | Marine | 74.820 | 17.000 | 2003/2004 | surface sediment | 0-1 | 280 | 0.90 | 0.1 | 0.08 | -22.6 | NA | Knies et al.^[11]^ |
| 22 | Marine | 74.820 | 16.030 | 2003/2004 | surface sediment | 0-1 | 356 | 0.73 | 0.07 | 0.05 | -23.4 | NA | Knies et al.^[11]^ |
| 23 | Marine | 74.820 | 14.790 | 2003/2004 | surface sediment | 0-1 | 1507 | 0.95 | 0.14 | 0.12 | -22.2 | NA | Knies et al.^[11]^ |
| 24 | Marine | 75.640 | 12.920 | 2003/2004 | surface sediment | 0-1 | 1500 | 1.11 | 0.16 | 0.13 | -22.5 | NA | Knies et al.^[11]^ |
| 25 | Marine | 75.750 | 13.840 | 2003/2004 | surface sediment | 0-1 | 807 | 0.82 | 0.08 | 0.05 | -23.5 | NA | Knies et al.^[11]^ |
| 26 | Marine | 75.830 | 14.770 | 2003/2004 | surface sediment | 0-1 | 370 | 1.66 | 0.18 | 0.13 | -23.0 | NA | Knies et al.^[11]^ |
| 27 | Marine | 75.950 | 15.720 | 2003/2004 | surface sediment | 0-1 | 369 | 2.17 | 0.25 | 0.19 | -22.7 | NA | Knies et al.^[11]^ |
| 28 | Marine | 76.050 | 16.670 | 2003/2004 | surface sediment | 0-1 | 328 | 1.69 | 0.19 | 0.14 | -22.6 | NA | Knies et al.^[11]^ |
| 29 | Marine | 76.160 | 17.620 | 2003/2004 | surface sediment | 0-1 | 309 | 2.31 | 0.27 | 0.20 | -22.9 | NA | Knies et al.^[11]^ |
| 30 | Marine | 76.220 | 18.580 | 2003/2004 | surface sediment | 0-1 | 257 | 2.24 | 0.26 | 0.19 | -22.6 | NA | Knies et al.^[11]^ |
| 31 | Marine | 76.310 | 19.570 | 2003/2004 | surface sediment | 0-1 | 258 | 2.66 | 0.31 | 0.23 | -22.7 | NA | Knies et al.^[11]^ |
| 32 | Marine | 76.380 | 20.580 | 2003/2004 | surface sediment | 0-1 | 228 | 2.40 | 0.28 | 0.21 | -22.8 | NA | Knies et al.^[11]^ |
| 33 | Marine | 76.470 | 21.600 | 2003/2004 | surface sediment | 0-1 | 262 | 2.62 | 0.3 | 0.22 | -22.7 | NA | Knies et al.^[11]^ |
| 34 | Marine | 71.750 | 22.000 | 2003/2004 | surface sediment | 0-1 | 356 | 0.76 | 0.11 | 0.09 | -21.8 | NA | Knies et al.^[11]^ |
| 35 | Marine | 71.620 | 21.070 | 2003/2004 | surface sediment | 0-1 | 319 | 0.60 | 0.09 | 0.07 | -21.8 | NA | Knies et al.^[11]^ |
| 36 | Marine | 71.600 | 20.860 | 2003/2004 | surface sediment | 0-1 | 320 | 0.80 | 0.12 | 0.10 | -21.4 | NA | Knies et al.^[11]^ |
| 37 | Marine | 71.600 | 21.190 | 2003/2004 | surface sediment | 0-1 | 335 | 0.58 | 0.09 | 0.07 | -21.8 | NA | Knies et al.^[11]^ |
| 39 | Marine | 71.340 | 20.190 | 2003/2004 | surface sediment | 0-1 | 234 | 0.75 | 0.13 | 0.11 | -21.2 | NA | Knies et al.^[11]^ |
| 40 | Marine | 71.180 | 19.560 | 2003/2004 | surface sediment | 0-1 | 225 | 0.83 | 0.13 | 0.11 | -21.2 | NA | Knies et al.^[11]^ |
| 41 | Marine | 71.030 | 18.950 | 2003/2004 | surface sediment | 0-1 | 199 | 0.37 | 0.05 | 0.05 | -21.4 | NA | Knies et al.^[11]^ |
| 42 | Marine | 70.870 | 18.340 | 2003/2004 | surface sediment | 0-1 | 173 | 0.43 | 0.06 | 0.05 | -22.0 | NA | Knies et al.^[11]^ |
| 44 | Marine | 70.550 | 17.140 | 2003/2004 | surface sediment | 0-1 | 706 | 0.20 | 0.03 | 0.02 | -21.3 | NA | Knies et al.^[11]^ |
| 627 | Marine | 72.320 | 24.060 | 2003/2004 | surface sediment | 0-1 | 264 | 0.62 | 0.09 | 0.07 | -22.3 | NA | Knies et al.^[11]^ |
| 629 | Marine | 73.010 | 24.250 | 2003/2004 | surface sediment | 0-1 | 404 | 1.39 | 0.18 | 0.14 | -22.9 | NA | Knies et al.^[11]^ |
| 633 | Marine | 74.340 | 24.690 | 2003/2004 | surface sediment | 0-1 | 373 | 1.26 | 0.13 | 0.08 | -23.5 | NA | Knies et al.^[11]^ |
| 635 | Marine | 75.000 | 24.940 | 2003/2004 | surface sediment | 0-1 | 182 | 2.45 | 0.3 | 0.24 | -22.9 | NA | Knies et al.^[11]^ |
| 639 | Marine | 75.570 | 27.900 | 2003/2004 | surface sediment | 0-1 | 263 | 2.74 | 0.34 | 0.28 | -22.6 | NA | Knies et al.^[11]^ |
| 643 | Marine | 76.490 | 29.910 | 2003/2004 | surface sediment | 0-1 | 291 | 2.24 | 0.27 | 0.22 | -22.8 | NA | Knies et al.^[11]^ |
| 645 | Marine | 75.860 | 29.460 | 2003/2004 | surface sediment | 0-1 | 296 | 2.20 | 0.21 | 0.15 | -24.4 | NA | Knies et al.^[11]^ |
| 651 | Marine | 74.640 | 26.080 | 2003/2004 | surface sediment | 0-1 | 317 | 2.08 | 0.26 | 0.21 | -22.7 | NA | Knies et al.^[11]^ |
| 653 | Marine | 73.970 | 25.810 | 2003/2004 | surface sediment | 0-1 | 441 | 1.73 | 0.22 | 0.18 | -22.7 | NA | Knies et al.^[11]^ |
| 655 | Marine | 73.310 | 25.540 | 2003/2004 | surface sediment | 0-1 | 412 | 1.53 | 0.2 | 0.16 | -22.8 | NA | Knies et al.^[11]^ |
| 661 | Marine | 71.370 | 22.760 | 2003/2004 | surface sediment | 0-1 | 408 | 0.71 | 0.11 | 0.09 | -22.1 | NA | Knies et al.^[11]^ |
| 665 | Marine | 72.170 | 28.410 | 2003/2004 | surface sediment | 0-1 | 289 | 0.78 | 0.11 | 0.09 | -22.5 | NA | Knies et al.^[11]^ |
| 669 | Marine | 73.500 | 29.150 | 2003/2004 | surface sediment | 0-1 | 414 | 1.37 | 0.18 | 0.14 | -22.5 | NA | Knies et al.^[11]^ |
| 673 | Marine | 74.670 | 32.490 | 2003/2004 | surface sediment | 0-1 | 165 | 1.28 | 0.13 | 0.09 | -23.2 | NA | Knies et al.^[11]^ |
| 677 | Marine | 75.970 | 33.730 | 2003/2004 | surface sediment | 0-1 | 276 | 1.91 | 0.18 | 0.12 | -23.7 | NA | Knies et al.^[11]^ |
| 679 | Marine | 76.620 | 34.450 | 2003/2004 | surface sediment | 0-1 | 193 | 1.82 | 0.23 | 0.18 | -23.5 | NA | Knies et al.^[11]^ |
| 690 | Marine | 71.020 | 30.960 | 2003/2004 | surface sediment | 0-1 | 283 | 0.39 | 0.05 | 0.03 | -23.8 | NA | Knies et al.^[11]^ |
| 1239 | Norwegian Sea | 74.460 | 20.830 | summer 2001 | surface sediment | 0-1 | 178 | 1.70 | 0.2 | 0.16 | -22.7 | NA | Winkelmann and Knies^[20]^ |
| 1240 | Norwegian Sea | 74.810 | 19.180 | summer 2001 | surface sediment | 0-1 | 94 | 0.53 | 0.07 | 0.05 | -22.6 | NA | Winkelmann and Knies^[20]^ |
| 1241 | Norwegian Sea | 74.820 | 17.580 | summer 2001 | surface sediment | 0-1 | 297 | 2.14 | 0.29 | 0.25 | -22.3 | NA | Winkelmann and Knies^[20]^ |
| 1242 | Norwegian Sea | 75.500 | 13.330 | summer 2001 | surface sediment | 0-1 | 1297 | 1.07 | 0.16 | 0.14 | -22.2 | NA | Winkelmann and Knies^[20]^ |
| 1243 | Norwegian Sea | 76.000 | 16.590 | summer 2001 | surface sediment | 0-1 | 333 | 1.82 | 0.23 | 0.18 | -22.8 | NA | Winkelmann and Knies^[20]^ |
| 1244 | Storfjorden | 77.950 | 19.170 | summer 2001 | surface sediment | 0-1 | 96 | 2.17 | 0.24 | 0.14 | -25.2 | NA | Winkelmann and Knies^[20]^ |
| 1245 | Storfjorden | 77.500 | 19.130 | summer 2001 | surface sediment | 0-1 | 180 | 2.37 | 0.27 | 0.17 | -24.2 | NA | Winkelmann and Knies^[20]^ |
| 1246 | Marine | 76.940 | 19.440 | summer 2001 | surface sediment | 0-1 | 153 | 2.07 | 0.23 | 0.14 | -23.8 | NA | Winkelmann and Knies^[20]^ |
| 1249 | Hornsund | 76.950 | 15.250 | summer 2001 | surface sediment | 0-1 | 156 | 1.91 | 0.23 | 0.14 | -23.6 | NA | Winkelmann and Knies^[20]^ |
| 1250 | Hornsund | 76.980 | 15.760 | summer 2001 | surface sediment | 0-1 | 228 | 1.65 | 0.18 | 0.10 | -23.9 | NA | Winkelmann and Knies^[20]^ |
| 1251 | van Mijenfjorden | 77.750 | 14.920 | summer 2001 | surface sediment | 0-1 | 115 | 1.84 | 0.17 | 0.08 | -24.6 | NA | Winkelmann and Knies^[20]^ |
| 1254 | van Mijenfjorden | 77.830 | 16.590 | summer 2001 | surface sediment | 0-1 | 76 | 2.03 | 0.14 | 0.04 | -24.8 | NA | Winkelmann and Knies^[20]^ |
| 1255 | van Mijenfjorden | 77.720 | 15.190 | summer 2001 | surface sediment | 0-1 | 83 | 1.74 | 0.15 | 0.07 | -24.8 | NA | Winkelmann and Knies^[20]^ |
| 1258 | van Mijenfjorden | 77.830 | 15.690 | summer 2001 | surface sediment | 0-1 | 43 | 1.72 | 0.13 | 0.05 | -25.1 | NA | Winkelmann and Knies^[20]^ |
| 1260 | Marine | 77.630 | 14.210 | summer 2001 | surface sediment | 0-1 | 162 | 1.77 | 0.23 | 0.16 | -23.2 | NA | Winkelmann and Knies^[20]^ |
| 1261 | Marine | 77.390 | 10.600 | summer 2001 | surface sediment | 0-1 | 1291 | 0.89 | 0.12 | 0.08 | -23.4 | NA | Winkelmann and Knies^[20]^ |
| 1262 | Marine | 77.360 | 11.270 | summer 2001 | surface sediment | 0-1 | 603 | 0.82 | 0.09 | 0.05 | -24.4 | NA | Winkelmann and Knies^[20]^ |
| 1263 | Marine | 77.200 | 12.930 | summer 2001 | surface sediment | 0-1 | 196 | 1.96 | 0.24 | 0.16 | -23.5 | NA | Winkelmann and Knies^[20]^ |
| 1264 | Marine | 77.540 | 12.600 | summer 2001 | surface sediment | 0-1 | 103 | 1.53 | 0.19 | 0.13 | -23.2 | NA | Winkelmann and Knies^[20]^ |
| 1265 | Isfjorden | 78.370 | 16.370 | summer 2001 | surface sediment | 0-1 | 87 | 1.88 | 0.17 | 0.09 | -25.2 | NA | Winkelmann and Knies^[20]^ |
| 1266 | Isfjorden | 78.360 | 15.260 | summer 2001 | surface sediment | 0-1 | 256 | 2.24 | 0.28 | 0.20 | -23.7 | NA | Winkelmann and Knies^[20]^ |
| 1267 | Isfjorden | 78.150 | 13.830 | summer 2001 | surface sediment | 0-1 | 416 | 2.68 | 0.37 | 0.29 | -22.9 | NA | Winkelmann and Knies^[20]^ |
| 1269 | Marine | 78.370 | 12.300 | summer 2001 | surface sediment | 0-1 | 169 | 2.00 | 0.25 | 0.16 | -23.8 | NA | Winkelmann and Knies^[20]^ |
| 1270 | Marine | 78.080 | 12.300 | summer 2001 | surface sediment | 0-1 | 259 | 2.41 | 0.31 | 0.22 | -22.8 | NA | Winkelmann and Knies^[20]^ |
| 1272 | Marine | 78.250 | 8.820 | summer 2001 | surface sediment | 0-1 | 1400 | 1.20 | 0.15 | 0.11 | -22.9 | NA | Winkelmann and Knies^[20]^ |
| 1273 | Marine | 78.250 | 9.320 | summer 2001 | surface sediment | 0-1 | 600 | 0.85 | 0.08 | 0.04 | -24.0 | NA | Winkelmann and Knies^[20]^ |
| 1274 | Marine | 78.250 | 9.390 | summer 2001 | surface sediment | 0-1 | 430 | 0.89 | 0.1 | 0.05 | -24.2 | NA | Winkelmann and Knies^[20]^ |
| 1275 | Marine | 78.250 | 10.210 | summer 2001 | surface sediment | 0-1 | 297 | 2.21 | 0.26 | 0.19 | -23.0 | NA | Winkelmann and Knies^[20]^ |
| 1276 | Marine | 78.570 | 10.340 | summer 2001 | surface sediment | 0-1 | 131 | 2.14 | 0.31 | 0.25 | -22.4 | NA | Winkelmann and Knies^[20]^ |
| 1277 | Marine | 78.580 | 9.440 | summer 2001 | surface sediment | 0-1 | 404 | 1.44 | 0.18 | 0.13 | -23.0 | NA | Winkelmann and Knies^[20]^ |
| 1278 | Marine | 78.580 | 9.030 | summer 2001 | surface sediment | 0-1 | 601 | 1.13 | 0.13 | 0.09 | -23.1 | NA | Winkelmann and Knies^[20]^ |
| 1279 | Marine | 78.610 | 7.820 | summer 2001 | surface sediment | 0-1 | 1203 | 1.36 | 0.18 | 0.13 | -22.9 | NA | Winkelmann and Knies^[20]^ |
| 1280 | Marine | 78.610 | 8.700 | summer 2001 | surface sediment | 0-1 | 787 | 1.31 | 0.16 | 0.11 | -23.1 | NA | Winkelmann and Knies^[20]^ |
| 1281 | Marine | 78.720 | 9.830 | summer 2001 | surface sediment | 0-1 | 106 | 1.29 | 0.17 | 0.13 | -22.4 | NA | Winkelmann and Knies^[20]^ |
| 1282 | Marine | 78.950 | 6.820 | summer 2001 | surface sediment | 0-1 | 1400 | 1.18 | 0.16 | 0.12 | -22.9 | NA | Winkelmann and Knies^[20]^ |
| 1283 | Marine | 78.950 | 8.310 | summer 2001 | surface sediment | 0-1 | 812 | 1.22 | 0.16 | 0.12 | -22.9 | NA | Winkelmann and Knies^[20]^ |
| 1285 | Marine | 78.820 | 9.620 | summer 2001 | surface sediment | 0-1 | 91 | 1.96 | 0.22 | 0.16 | -22.7 | NA | Winkelmann and Knies^[20]^ |
| 1286 | Marine | 78.870 | 11.330 | summer 2001 | surface sediment | 0-1 | 159 | 1.13 | 0.16 | 0.12 | -21.9 | NA | Winkelmann and Knies^[20]^ |
| 1287 | Kongsfjorden | 79.170 | 11.770 | summer 2001 | surface sediment | 0-1 | 364 | 0.97 | 0.13 | 0.11 | -21.9 | NA | Winkelmann and Knies^[20]^ |
| 1288 | Kongsfjorden | 78.980 | 11.820 | summer 2001 | surface sediment | 0-1 | 308 | 1.38 | 0.19 | 0.15 | -22.3 | NA | Winkelmann and Knies^[20]^ |
| 1289 | Marine | 79.030 | 10.840 | summer 2001 | surface sediment | 0-1 | 319 | 2.23 | 0.29 | 0.22 | -22.4 | NA | Winkelmann and Knies^[20]^ |
| 1290 | Marine | 78.950 | 9.620 | summer 2001 | surface sediment | 0-1 | 250 | 0.95 | 0.13 | 0.10 | -22.4 | NA | Winkelmann and Knies^[20]^ |
| 1292 | Marine | 79.060 | 9.330 | summer 2001 | surface sediment | 0-1 | 80 | 1.51 | 0.21 | 0.17 | -22.0 | NA | Winkelmann and Knies^[20]^ |
| PS2824 | Yermak Plateau | 77.580 | 34.680 | summer 1997 | surface sediment | 0-1 | 212 | 1.80 | 0.22 | 0.18 | -24.2 | NA | Schubert and Calvert^[21]^ |
| PS2827 | Yermak Plateau | 79.440 | 30.470 | summer 1997 | surface sediment | 0-1 | 335 | 1.60 | 0.21 | 0.17 | -23.8 | NA | Schubert and Calvert^[21]^ |
| PS2831 | Yermak Plateau | 81.100 | 16.980 | summer 1997 | surface sediment | 0-1 | 917 | 0.69 | 0.09 | 0.07 | -23.3 | NA | Schubert and Calvert^[21]^ |
| PS2832 | Yermak Plateau | 81.110 | 16.200 | summer 1997 | surface sediment | 0-1 | 2070 | 0.78 | 0.11 | 0.08 | -23.3 | NA | Schubert and Calvert^[21]^ |
| PS2834 | Yermak Plateau | 80.920 | 9.820 | summer 1997 | surface sediment | 0-1 | 1029 | 1.26 | 0.15 | 0.11 | -23.3 | NA | Schubert and Calvert^[21]^ |
| PS2835 | Yermak Plateau | 81.100 | 7.070 | summer 1997 | surface sediment | 0-1 | 891 | 1.13 | 0.14 | 0.10 | -23.5 | NA | Schubert and Calvert^[21]^ |
| PS2837 | Yermak Plateau | 81.230 | 2.420 | summer 1997 | surface sediment | 0-1 | 992 | 1.90 | 0.21 | 0.16 | -23.2 | NA | Schubert and Calvert^[21]^ |
| PS2839 | Yermak Plateau | 81.400 | -0.970 | summer 1997 | surface sediment | 0-1 | 2905 | 1.37 | 0.15 | 0.11 | -23.5 | NA | Schubert and Calvert^[21]^ |
| PS2847 | Yermak Plateau | 81.870 | -4.540 | summer 1997 | surface sediment | 0-1 | 4268 | 0.92 | 0.11 | 0.07 | -23.6 | NA | Schubert and Calvert^[21]^ |
| PS2853 | Yermak Plateau | 82.320 | 3.710 | summer 1997 | surface sediment | 0-1 | 2061 | 0.90 | 0.1 | 0.07 | -23.5 | NA | Schubert and Calvert^[21]^ |
| PS2855 | Yermak Plateau | 82.050 | 5.290 | summer 1997 | surface sediment | 0-1 | 1455 | 1.54 | 0.17 | 0.13 | -23.0 | NA | Schubert and Calvert^[21]^ |
| PS2856 | Yermak Plateau | 81.990 | 5.750 | summer 1997 | surface sediment | 0-1 | 929 | 1.45 | 0.16 | 0.11 | -24.1 | NA | Schubert and Calvert^[21]^ |
| PS2857 | Yermak Plateau | 81.900 | 7.910 | summer 1997 | surface sediment | 0-1 | 824 | 1.21 | 0.15 | 0.11 | -23.5 | NA | Schubert and Calvert^[21]^ |
| PS2860 | Yermak Plateau | 81.580 | 11.850 | summer 1997 | surface sediment | 0-1 | 1993 | 1.50 | 0.16 | 0.12 | -22.9 | NA | Schubert and Calvert^[21]^ |
| PS2862 | Yermak Plateau | 80.580 | 11.790 | summer 1997 | surface sediment | 0-1 | 1035 | 1.44 | 0.2 | 0.15 | -23.1 | NA | Schubert and Calvert^[21]^ |
| PS2865 | Yermak Plateau | 80.500 | 10.490 | summer 1997 | surface sediment | 0-1 | 822 | 1.74 | 0.19 | 0.13 | -24.2 | NA | Schubert and Calvert^[21]^ |

Table S5. Compilation data of δ^13^C_org_ end-member values reported in previous studies conducted around Svalbard. NA denotes “not available”. Note that the end-member values indicated as ‘this study’ were obtained following the approach by Knies et al.^[21]^, using the newly generated (Table S1) as well as compiled data from the literature (Table S4).

| **Study area** | **Terrestrial end-member  δ^13^C** (‰) | **Marine end-member  δ^13^C** (‰) | **Sample type** | **Reference** |
| --- | --- | --- | --- | --- |
| around Svalbard | −25.8 | −20.5 | surface sediments | This study |
| Yermak Plateau | −27.1 | −21.4 | surface sediments | Schubert and Calvert^[21]^ |
| Northern Norwegian continental shelf | −27.0 | −20.3 | surface sediments | Knies et al.^[19]^ |
| around Svalbard | −26.8 | −20.6 | surface sediments | Winkelmann and Knies^[20]^ |
| around Svalbard + Western Barents Sea region | −26.1 | −20.1 | surface sediments | Knies et al.^[11]^ |
| Wijdefjorden | −26.8 | −20.6 | surface sediments | Kim et al.^[14]^ |
| Woodfjorden | NA | NA | NA | NA |
| Dicksonfjorden | −26.8 | −20.6 | surface sediments | Kim et al.^[14]^ |
| Isfjorden | −28.5 | −22.5 | suspended particulate matter | Holding et al.^[9]^ |
| van Mijenfjorden | NA | NA | NA | NA |
| Hornsund | −26.8 | −20.6 | surface sediments | Kim et al.^[14]^ |
| Hambergbukta | NA | NA | NA | NA |
| Kongsfjorden | −22.5 | −24.0 | surface sediments | Kumar et al^[17]^ |
| Kongsfjorden | −26.1 | NA | Soil | Kim et al.^[4]^ |
| Kongsfjorden | −22.6 | −22.3 | IRD, surface sediments | Kim et al.^[4]^ |
| Kongsfjorden | −26.7 | −19.0 | suspended particulate matter, marine phytobenthos and plankton | Kuliński et al.^[10]^ |

Table S6. OC end-member values utilized for estimating the relative proportion of OC using the MC analyses. SD denotes “standard deviation”.

| **End-member** | **Δ^14^C_org_**  **(**‰) | **SD (±1**σ**)** | **δ^13^C_org_**  **(**‰) | **SD (±1**σ**)** | **(Ad/Al)v** | **SD**  **(±1**σ**)** |
| --- | --- | --- | --- | --- | --- | --- |
| Petrogenic OC | −1000.0 | 0.0 | −25.74 | 1.89 | 0.49 | 0.16 |
| Soil-derived OC | −344.4 | 358.9 | −25.48 | 2.25 | 1.37 | 1.47 |
| Plant-derived OC | 9.1 | 6.4 | −31.38 | 1.63 | 0.33 | 0.09 |
| Marine OC | 5.2 | 6.3 | −19.9 | 0.20 | 0.00 | 0.00 |


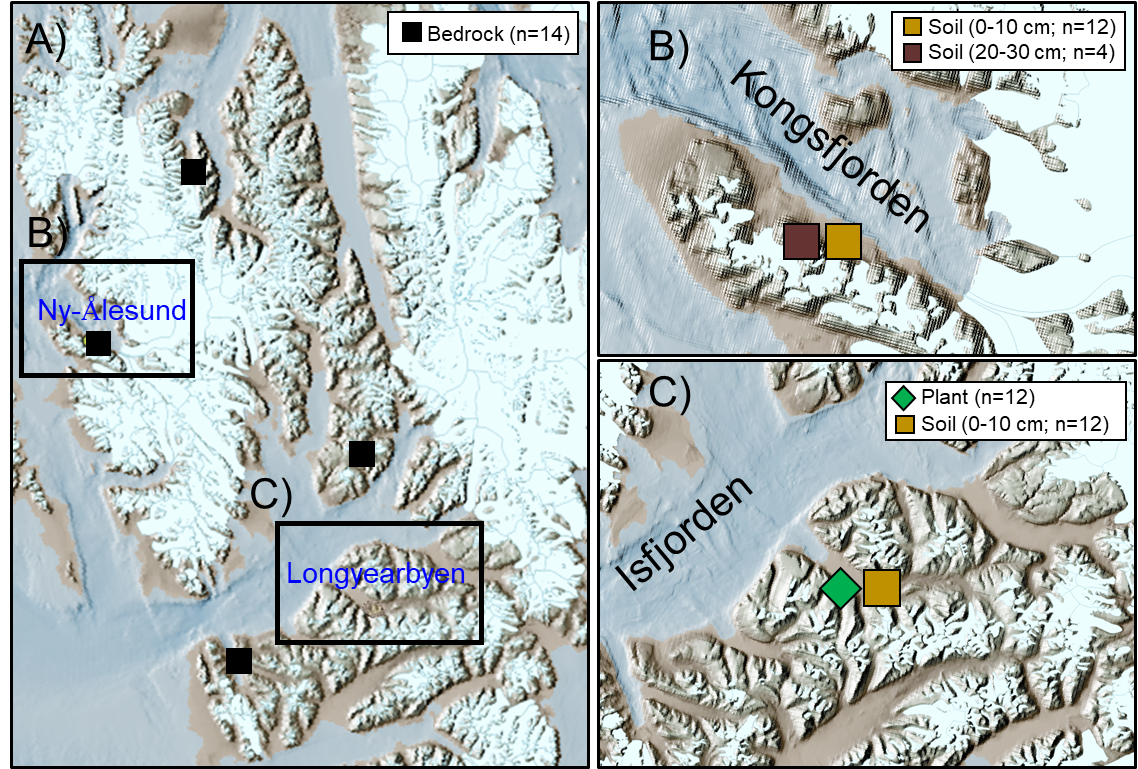


Figure S1. Maps showing the sampling sites for bedrocks in the western part of the Svalbard archipelago (A), as well as the sampling sites for plants and soils around Ny-Ålesund (B) and Longyearbyen (C). The white areas on the map indicate the current glacier coverage. The maps were generated using QGIS v3.14 (https://qgis.org/en/site/forusers/visualchangelog314/) based on IBCAOv4^[22]^ (https://www.ngdc.noaa.gov/mgg/bathymetry/arctic/). See also Supplementary Table S5 for detailed information on the samples.


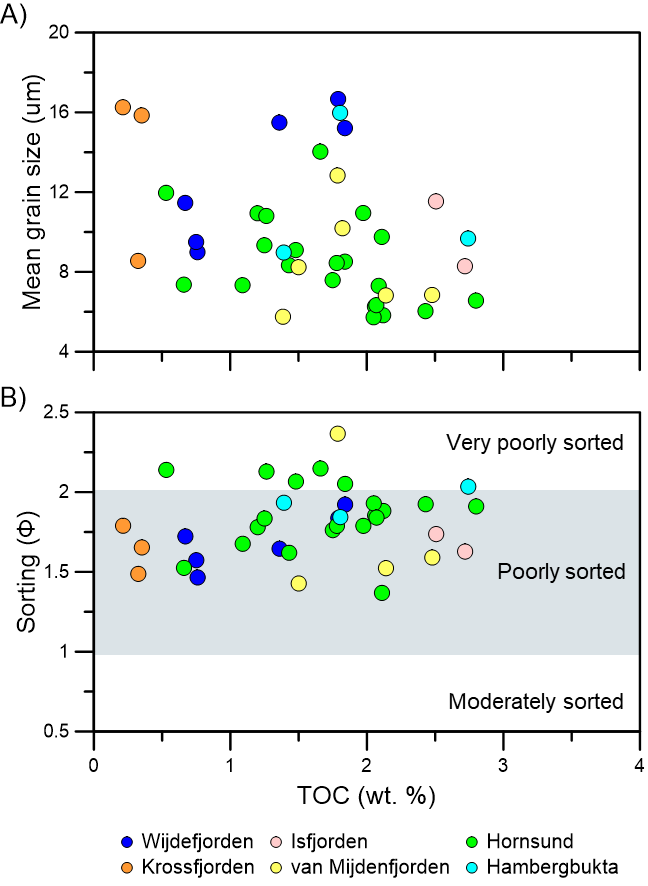


Figure S2. Scatter plots of the A) mean grain size (µm) and B) sorting (Ф) versus TOC contents (wt. %).

Figure S3. Scatter plots of TOC versus N_tot_ and N_org_ contents in A) Wijdefjorden, B) Woodfjorden, C) Dicksonfjorden, D) Isfjorden, E) van Mijenfjorden, F) Hornsund, G) Hamberbukta, and H) Krossfjorden. Note that the open and filled circles correspond to the left and right y-axes, respectively.

Figure S4. Scatter plots of δ^13^C_org_ versus N_tot_/TOC and N_org_/TOC ratios in A) Wijdefjorden, B) Woodfjorden, C) Dicksonfjorden, D) Isfjorden, E) van Mijenfjorden, F) Hornsund, G) Hamberbukta, and H) Krossfjorden. Note that the open and filled circles correspond to the left and right y-axes, respectively.

Figure S5. Scatter plots of N_org_/TOC ratio versus δ^13^C_org_.


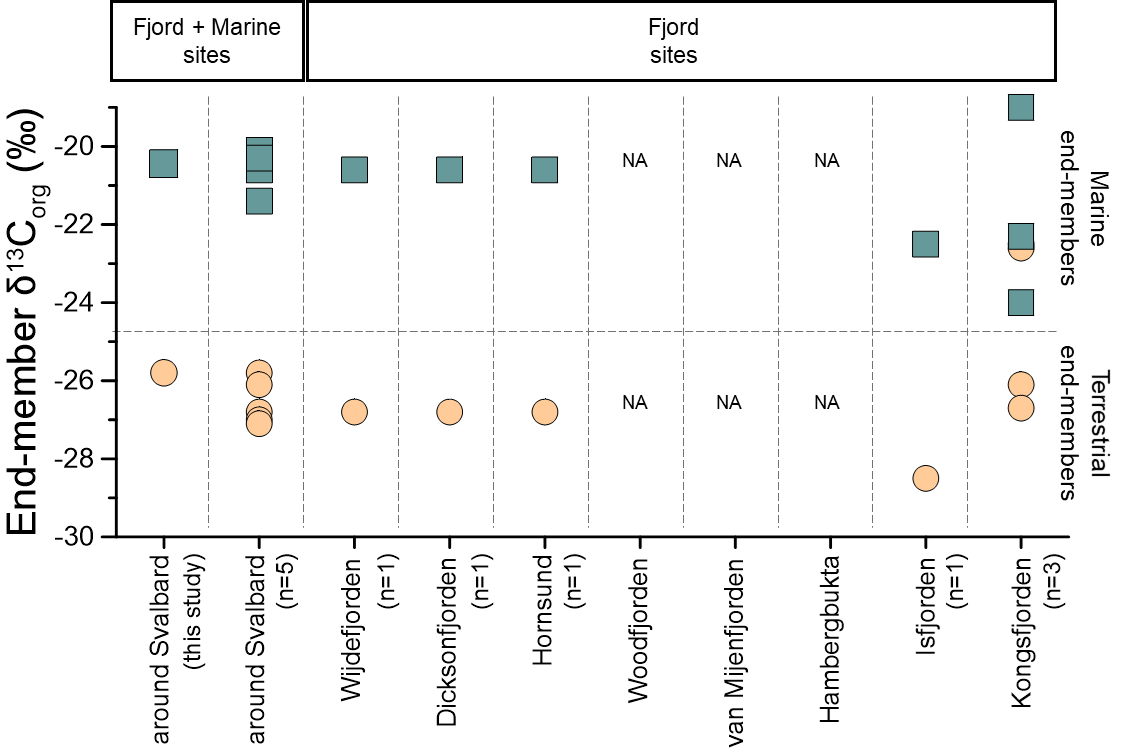


Figure S6. Compilation data of marine and terrestrial end-member δ^13^C_org_ considered in this study (see also Supplementary Table S5). “NA” denotes “not available”. The green squares represent the end-members of marine OC, while the pale orange circles represent the end-members of terrestrial OC. Note that the end-member values indicated as ‘this study’ were obtained following the approach by Knies et al.^[11]^ (see Fig. 2), using the newly generated (Table S1) as well as compiled data from the literature (Table S4).


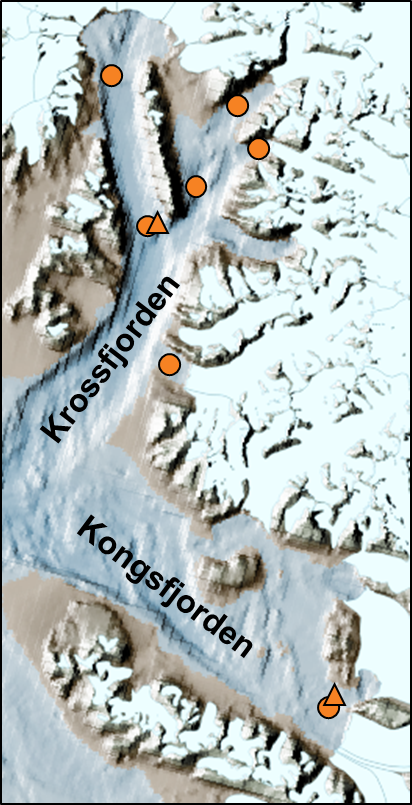


Figure S7. Map of the sampling sites for surface sediments (orange filled circles) and IRD (orange filled triangles) in the vicinity of glaciers in Kross/Kongsfjorden. The white areas on the map indicate the current glacier coverage. The map was generated using QGIS v3.14 (https://qgis.org/en/site/forusers/visualchangelog314/) based on IBCAOv4^[22]^ (https://www.ngdc.noaa.gov/mgg/bathymetry/arctic/). See also Supplementary Tables S1 and S4 for detailed information on the samples.


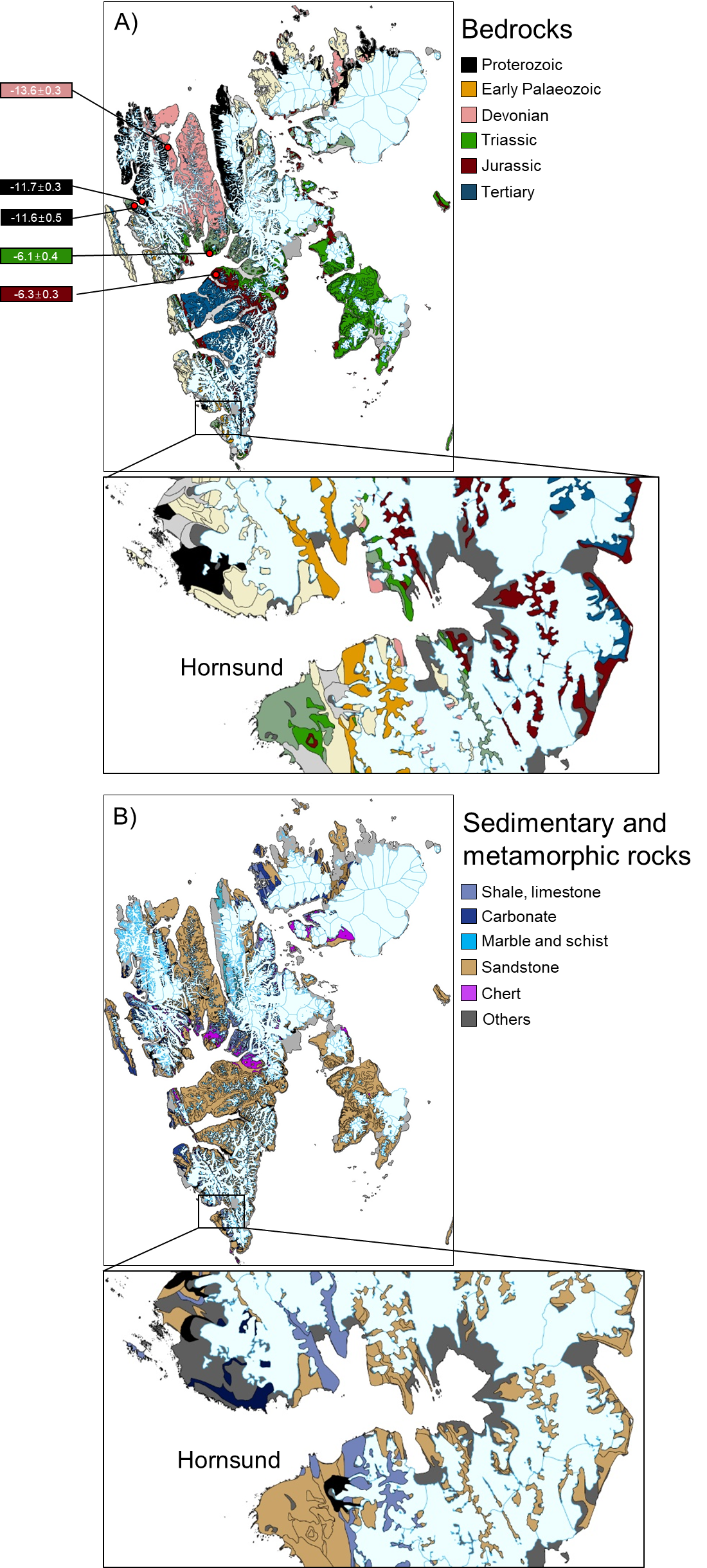


Figure S8. Distribution of A) bedrocks and B) sedimentary/metamorphic rocks in Svalbard. Note that the detrital Nd isotopes of the bedrocks in A) are from Jang et al.^[3]^. The white areas on the map indicate the current glacier coverage. The maps were generated using QGIS v3.14 (https://qgis.org/en/site/forusers/visualchangelog314/) based on IBCAOv4^[22]^ (https://www.ngdc.noaa.gov/mgg/bathymetry/arctic/) and data from Dallmann and Elvevold^[23]^.


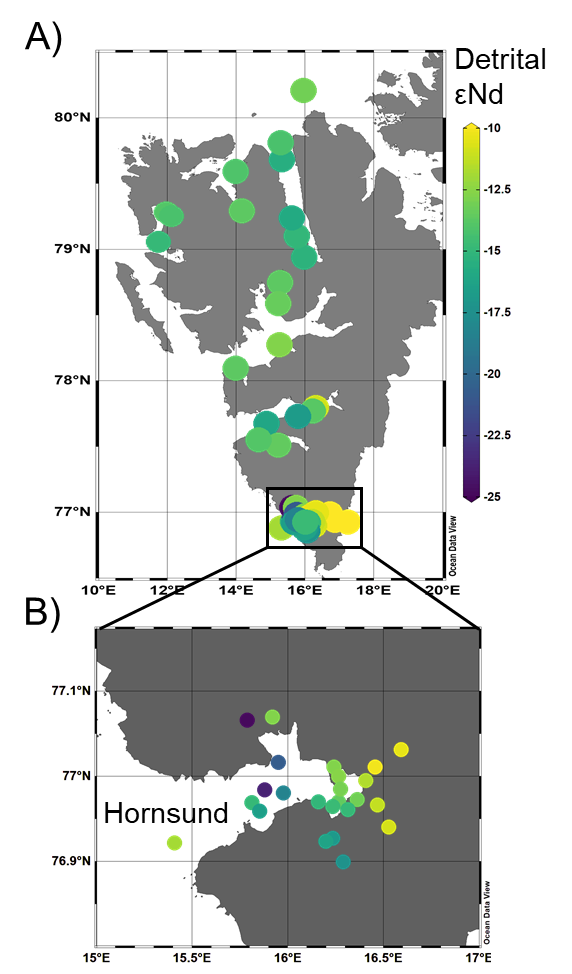


Figure S9. A) Spatial distribution of detrital Nd isotopes for Svalbard surface sediments and B) the enlarged part of Hornsund. The maps were generated with the Ocean Data View version 5.6.3 (GlobalHR, https://odv.awi.de).

Figure S10. Scatter plots of TOC_samples_ (wt. %) versus TOC_sample_*Fm_sample_ obtained in this study.


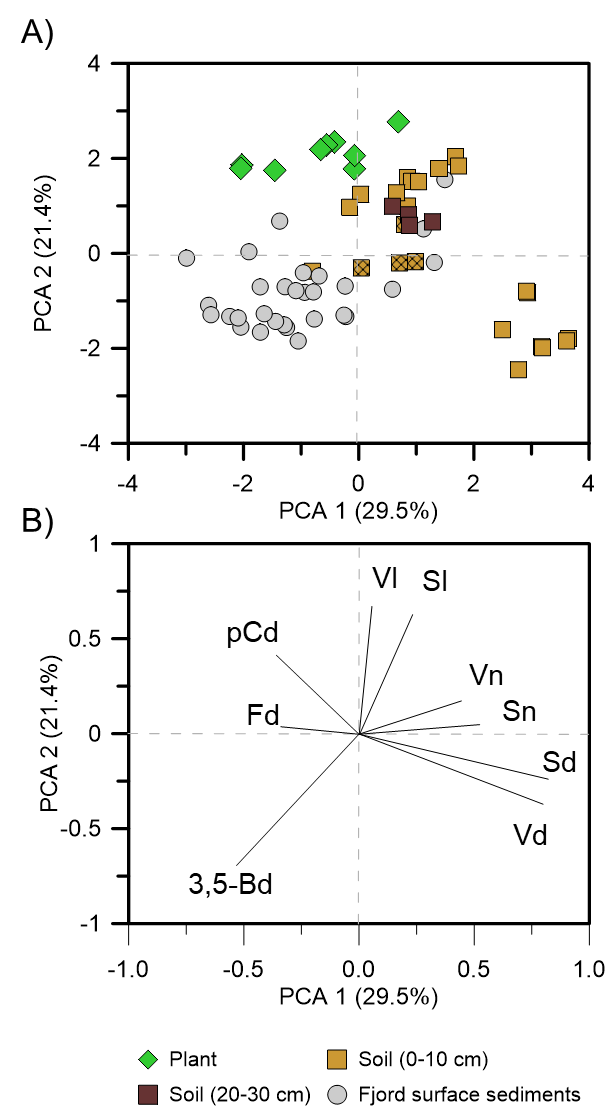


Figure S11. Results of principal component analysis (PCA) based on lignin phenol data obtained from A) plants, soils, and fjord surface sediments, and B) variables. Note that the soil samples collected at a depth of 0-10 cm, marked with hatching, were specifically obtained from areas in the vicinity of retreating land-terminating glaciers. The abbreviations used for the lignin phenols are as follows: Vl (vanillin), Vn (acetovanillone), Vd (vanillic acid), Sl (syringaldehyde), Sn (acetosyringone), Sd (syringic acid), pCd (p-coumaric acid), Fd (ferulic acid), and 3,5-Bd (3,5-dihydroxybenzoic acid).

Figure S12. Variations in relative OC fractions: A) petrogenic OC (%), B) soil-derived OC (%), C) plant-derived OC (%), and D) marine OC (%) calculated using the Monte Carlo (MC) approach. Note that no samples from the Woodfjorden were available for the Δ^14^C_org_ and lignin phenol analyses.

**Reference**

1. Vonk, J. E. *et al*. Activation of old carbon by erosion of coastal and subsea permafrost in Arctic Siberia. *Nature* **489**, 137–140. (2012).

2. Jong, D. *et al*. Contrasts in dissolved, particulate, and sedimentary organic carbon from the Kolyma River to the East Siberian Shelf. *Biogeosciences* **20**, 271–294 (2023).

3. Jang, K. *et al.* Neodymium isotope constraints on chemical weathering and past glacial activity in Svalbard. *Earth and Planetary Science Letters* **542**, 116319 (2020).

4. Kim, J.-H. *et al.* Large ancient organic matter contributions to Arctic marine sediments (Svalbard). *Limnology and Oceanography* **56**, 1463–1474 (2011).

5. Kusch, S. *et al*. Permafrost organic carbon turnover and export into a high-arctic fjord: A case study from Svalbard using compound-specific ^14^C Analysis. *Journal of Geophysical Research: Biogeosciences* **126**, e2020JG006008 (2021).

6. Broecker W. S. *et al*. Natural radiocarbon in the Atlantic Ocean. *Journal of Geophysical Research* **65**, 2903–2931 (1960).

7. Graven, H., Keeling, R. F. & Rogelj, J. Changes to carbon isotopes in atmospheric CO_2_ over the industrial era and into the future. *Global Biogeochemical Cycles* **34**, e2019GB006170 (2020).

8. Levin, I. *et al*. Radiocarbon in global tropospheric carbon dioxide. *Radiocarbon* **64**, 781–791 (2022).

9. Holding, J. M. *et al*. Autochthonous and allochthonous contributions of organic carbon to microbial food webs in Svalbard fjords. *Limnology and Oceanography* **62**, 1307–1323 (2017).

10. Kuliński, K. *et al*. Particulate organic matter sinks and sources in high Arctic fjord. *Journal of Marine Systems* **139**, 27–37 (2014).

11. Knies, J. & Martinez, P. Organic matter sedimentation in the western Barents Sea region: Terrestrial and marine contribution based on isotopic composition and organic nitrogen content. *Norwegian Journal of Geology* **89**, 79–89 (2009).

12. Ruben, M. *et al*. Fossil organic carbon utilization in marine Arctic fjord sediments by subsurface micro-organisms. *Nature Geoscience* **1**, 1–6 (2023).

13. Sanz-Martin, M. *et al*. Relationship between carbon- and oxygen-based primary productivity in the Arctic Ocean, Svalbard Archipelago. *Frontiers in Marine Science* 6, **468** (2019).

14. Kim, H. *et al.* Input of terrestrial organic matter linked to deglaciation increased mercury transport to the Svalbard fjords. *Scientific reports* **10**, 3446 (2020).

15. Kim, Y. J. *et al*. Chronological changes in soil biogeochemical properties of the glacier foreland of Midtre Lovenbreen, Svalbard, attributed to soil-forming factors. *Geoderma* **415**, 115777 (2022).

16. Jung, J. Y. *et al*. Soil organic carbon characteristics relating to geomorphology near Vestre Lovenbreen moraine in Svalbard. *Ecology and Environment* **37**, 69–79 (2014).

17. Kumar, V. *et al*. Evidence of anomalously low δ^13^C of marine organic matter in an Arctic fjord. *Scientific Reports* **6**, 1–9 (2016).

18. Koziorowska, K., Kuliński, K. & Pempkowiak, J. Sedimentary organic matter in two Spitsbergen fjords: Terrestrial and marine contributions based on carbon and nitrogen contents and stable isotopes composition. *Continental Shelf Research* **113**, 38–46 (2016).

19. Knies, J., Brookes, S. & Schubert, C. J. Re-assessing the nitrogen signal in continental margin sediments: New insights from the high northern latitudes. *Earth and Planetary Science Letters* **253**, 471–484 (2007).

20. Winkelmann, D. & Knies, J. Recent distribution and accumulation of organic carbon on the continental margin west off Spitsbergen. *Geochemistry, Geophysics, Geosystems* **6,** Q09012 (2005).

21. Schubert, C. J. & Calvert, S. E. Nitrogen and carbon isotopic composition of marine and terrestrial organic matter in Arctic Ocean sediments: implications for nutrient utilization and organic matter composition. *Deep-Sea Research I* **48**, 789–810 (2001).

22. Jakobsson, M. *et al*. The International Bathymetric Chart of the Arctic Ocean Version 4.0. *Scientific Data* **7**, 176 (2020).

23. Dallmann, W. & Elvevold, S. Bedrock Geology. Geoscience Atlas of Svalbard. *Re-port Series* **148**, Norsk Polarinstitutt, Tromsø, pp.133–173 (2015).
